# Transcriptome Response Mediated by Cold Stress in *Lotus japonicus*

**DOI:** 10.3389/fpls.2016.00374

**Published:** 2016-03-30

**Authors:** Pablo I. Calzadilla, Santiago J. Maiale, Oscar A. Ruiz, Francisco J. Escaray

**Affiliations:** Unidad de Biotecnología 1, Instituto de Investigaciones Biotecnológicas, Instituto Tecnológico de Chascomús, Universidad Nacional de San Martín, Consejo Nacional de Investigaciones Científicas y TécnicasChascomús, Argentina

**Keywords:** legume, RNA-Seq, cold stress, transcriptome, forage

## Abstract

Members of the *Lotus* genus are important as agricultural forage sources under marginal environmental conditions given their high nutritional value and tolerance of various abiotic stresses. However, their dry matter production is drastically reduced in cooler seasons, while their response to such conditions is not well studied. This paper analyzes cold acclimation of the genus by studying *Lotus japonicus* over a stress period of 24 h. High-throughput RNA sequencing was used to identify and classify 1077 differentially expressed genes, of which 713 were up-regulated and 364 were down-regulated. Up-regulated genes were principally related to lipid, cell wall, phenylpropanoid, sugar, and proline regulation, while down-regulated genes affected the photosynthetic process and chloroplast development. Together, a total of 41 cold-inducible transcription factors were identified, including members of the AP2/ERF, NAC, MYB, and WRKY families; two of them were described as putative novel transcription factors. Finally, DREB1/CBFs were described with respect to their cold stress expression profiles. This is the first transcriptome profiling of the model legume *L. japonicus* under cold stress. Data obtained may be useful in identifying candidate genes for breeding modified species of forage legumes that more readily acclimate to low temperatures.

## Introduction

Legumes (Fabaceae) are the primary source of plant protein for human consumption and in livestock feed, as well as key components of natural and agricultural ecosystems. Members of the *Lotus* genus, which includes about 120 species around the world, are particularly well known due to their elevated adaptability to marginal environmental conditions. This feature has made species of the *Lotus* genus popular as alternative forage in South America and Australia, as well as for dune revegetation and the reclamation of soil that has been burned or contaminated by heavy metals (Escaray et al., 2012). Within the genus, *L. japonicus* has become a model legume (Handberg and Stougaard, 1992), having been extensively used in abiotic stress (Díaz et al., 2010; Sainz et al., 2010; Babuin et al., 2014) and root nodulation studies (López et al., 2008; Li et al., 2014); this utility mainly results from particular genomic features that make it especially useful for recently developed functional genomics techniques (Sato et al., 2008; Fukai et al., 2012; Urbañski et al., 2012).

Even though forage *Lotus* species are considered cool season plants (Blumenthal and McGraw, 1999), dry matter production is in fact minimal during colder seasons at high latitudes (Bullard and Crawford, 1995; Halling et al., 2004), causing substantial economic losses (Wheeler et al., 2000; Thakur et al., 2010). It is therefore important for breeders to understand the mechanisms through which these plants tolerate cold and freezing. Previous research has focused on the cold stress response of *Arabidopsis* and has included both genetic and biochemical approaches (Mckown et al., 1996; Hannah et al., 2005; Kaplan et al., 2007). However, few studies have addressed legumes (Lucau-Danila et al., 2012; Dinari et al., 2013).

Plants have various ways in which they respond to and tolerate cold stress. These include changes in the composition, structure, and function of the plasma membrane, the synthesis of cryoprotectant molecules (soluble sugars and low-molecular-weight nitrogenous compounds, such as proline), and an increase in the scavenging activity of reactive oxygen species (ROS; Steponkus, 1984; Apel and Hirt, 2004; Wang et al., 2013). Cold temperatures also induce distinct secondary metabolic pathways. In particular, flavonoids have been proposed as a primary target of study for understanding this phenomenon; this large family of metabolites, synthesized via the phenylpropanoid pathway, has been related to photoprotection, cold hardiness, drought resistance, and antioxidative ability (Christie et al., 1994; Chalker-Scott, 1999).

Most cold stress responses are due to changes in gene expression, for which many transcription factors (TFs) have already been identified (Chinnusamy et al., 2007). The most well documented pathways involve a class of DREB/CBF TFs that are upstream regulated by several proteins, such as ICE1, MYB15, and ZAT12 (Shinozaki et al., 2003; Maruyama et al., 2009); the genes that are regulated through these pathways are collectively identified as *cor* genes. A large number of these genes encode proteins with known enzyme activity, specifically molecular chaperones and LEA proteins (Thomashow, 1999; Maruyama et al., 2009).

Next-generation sequencing has dramatically improved the efficiency of transcriptome data collection (Donà et al., 2013; Pang et al., 2013). High-throughput RNA sequencing (RNA-Seq) has been used in gene discovery and regulatory network studies, particularly in higher plants under stress (Deyholos, 2010; Kakumanu et al., 2012; Wang et al., 2013).

In this study, we describe the first transcriptome profiling by combining RNA-Seq with DREB1/CBF and *cor* gene expression analysis of the model legume *L. japonicus* after subjecting it to cold stress. To the best of our knowledge, this is the first report of *L. japonicus* response under low temperature stress. The results of this study can therefore guide the development of new agricultural forage practices and serve as a template for future studies that expand this understanding to other species within the genus.

## Materials and methods

### Plant materials and growth conditions

Seeds from the *L. japonicus* ecotype Gifu B-129 were scarified by stirring in pure sulfuric acid for 3 min, washed 10 times with sterile distilled water, and then sowed in Petri dishes containing 0.8% agar solution. Plates were incubated for 7 days in a growth chamber under a 16/8 h photoperiod at 24°C/21°C ± 2°C (day/night) and 55/65 ± 5% relative humidity. Light, at a photosynthetic flux density of 250 μmol m^−2^ s^−1^, was provided by F 40W Grolux fluorescent tubes. Seedlings were transferred to sterilized sand-perlite (2:1), placed in the previously used growth chamber under the same conditions, and irrigated with half-strenght Hoagland solution (Hoagland and Arnon, 1950).

Plants with 4–6 fully developed leaves, corresponding to roughly 3 weeks of development, were used in all experiments.

### Treatments and experimental design

The experimental design was completely randomized, with three biological repetitions per treatment. For both the control and stress tests, the 3 week-old seedlings were placed in a Percival E-30B (Percival Scientific, Perry, IA, USA) growth chamber under a 16/8 h photoperiod (day/night). Lighting conditions were identical to those used in Section Plant Materials and Growth Conditions Plants under cold stress treatment were kept at 9°C during daylight simulation and 5°C at night, whereas the controls were kept at conditions identical to Section Plant Materials and Growth Conditions.

For RNA-Seq, shoots were harvested after 1 complete 24 h cycle, immediately frozen in liquid N_2_, and stored at −80°C until their RNA was completely extracted. For DREB1/CBF and *cor* gene expression analysis, shoots were harvested after 0, 1, 3, 8, and 24 h of cold stress treatment.

### RNA extraction and expression analysis

Total RNA was extracted using a Plant Spectrum Total RNA Kit (Sigma) according to the manufacturer's instructions. RNA was checked for quality and quantified using agarose gel electrophoresis and spectrophotometric analysis. Total RNA samples (5–10 μg/sample) were shipped on dry ice to the Instituto de Agrobiotecnologia de Rosario (Rosario, Argentina) for RNA-Seq and analysis.

mRNA was purified using oligo-Dt and cDNA was synthesized. Samples were prepared for RNA sequencing as described in the Illumina TruSeq® RNA Sample Preparation Guide (July 2012). High-performance, paired-end (2 × 100 bp) sequencing was performed on an Illumina Hiseq 1500. Low-quality RNA-Seq reads (Qscore < Q30) were analyzed using FastQC (Version 0.11.2) and discarded (Andrews, 2010). Then, a total of 174520000 reads were aligned against the *L. japonicus* genome (Gene Model, Release 2.5; Sato et al., 2008) using TopHat (Version 2.0.12; Trapnell et al., 2012).

To identify differentially expressed genes, a pair-wise comparison between normalized gene expression values (FPKM) for both conditions was performed using a *t*-test at the 99.99% confidence level using Cufflinks (Version 2.2.1; Trapnell et al., 2012). Illumina reads generated from all 6 samples are available at the NCBI BioProject browser, accession number PRJNA288510, BioSample accessions SAMN03801565, SAMN03801566, SAMN03801567, SAMN03801568, SAMN03801569, and SAMN03801570.

The overall RNA extraction procedure was identical for relative quantification of DREB1/CBF and *cor* genes. The absence of DNA from the RNA samples was tested by null polymerase chain reaction (PCR) amplification of the universal rDNA primer pair ITS1/ITS4 (Escaray et al., 2014). Then, cDNA was synthesized from 3 μg of total RNA using M-MLV Reverse Transcriptase (Promega) and 100 pmol of the oligo-Dt primers, as per the supplier's instructions.

### RNA-Seq transcript analysis

An MA-plot was constructed using R (Version 3.1.3) for Windows, which contained both the CummeRbund and ggplot2 packages (Team, 2014). All transcripts were used to make the plot.

Gene Ontology (GO) enrichment analysis was carried out to reveal the biological processes differentially expressed under the stress conditions, using Fisher's exact test against the *L. japonicus* entry in the legumeIP database (Al-Shahrour et al., 2004; Li et al., 2012). Blast2GO software (https://www.blast2go.com/) was used and applied only for those transcripts that showed significant differential expression, defined, with respect to fold change (FC), as those for which log_2_FC ≥ |2| (Conesa et al., 2005).

Identification of the Kyoto Encyclopedia of Genes and Genomes (KEGG) metabolic pathways modified under low temperature treatment was used to corroborate the GO enrichment analysis (Kanehisa et al., 2010). KEGG annotation was done with the KEGG Automatic Annotation Server (http://www.genome.jp/kaas-bin/kaas_main), again only for those transcripts for which log_2_ FC ≥ |2| (Moriya et al., 2007). Classification was also performed using the LegumeIP database (Li et al., 2012).

Up-regulated TFs were identified and classified through PlantTFDB (http://planttfdb.cbi.pku.edu.cn/) and the *L. japonicus* entry in the Legume IP database (Li et al., 2012; Jin et al., 2014). Annotation information was obtained from GenBank (http://www.ncbi.nlm.nih.gov/genbank/, NCBI-GenBank Release 205.0, Dec, 2014www.ncbi.nlm.nih.gov/genbank/, NCBI-GenBank Release 205.0, Dec, 2014), LegumeIP [http://plantgrn.noble.org/LegumeIP/, LegumeIP v2 (beta version)] and the KEGG database (http://www.genome.jp/kegg, Release 74.0, April 1, 2015; Kanehisa et al., 2010; Li et al., 2012). Transcripts not functionally annotated by one or more of the above methods were assigned functional annotations based on a BLASTX search vs. the Genbank NR protein database for *E*-values less than 10^−3^; the lowest *E*-value sequences were chosen as representative.

To validate the results obtained from RNA-Seq, the expression data of 10 randomly chosen genes were analyzed by quantitative real time PCR (qRT-PCR; Supplementary Table 1). A linear regression analysis of RNA-Seq and qRT-PCR values was completed, resulting in *R*^2^ = 0.71 (Pearson's correlation r = 0.84; Supplementary Figure 1).

### qRT-PCR

To validate the RNA-Seq analyses and to quantify the relative expression of DREB1/CBF and *cor* genes, primers were designed with the help of Primer3 software (Untergasser et al., 2012). The primer pairs are given in Supplementary Table 1. An aliquot of 5 μL of 1:8 diluted cDNA was used in the qRT-PCR reactions, made using 15 μL of the FastStart Universal SYBR-Green Master Mix (Rox, Roche) and 2.5 pmol of each primer. Three biological replicates, every one accompanied by two technical replicates, were performed for each sample and gene. Cycling parameters were used that consisted of an initial step of 95°C for 10 min and a two-step cycle of 95°C for 30 s and 60°C for 1 min, repeated 40 times. This was followed by the dissociation protocol. Amplifications were performed on an Mx3005P qPCR apparatus (Stratagene, CA, USA). The average threshold cycle was determined for each transcript. Gene quantification was determined based on the relative expression of the target gene vs. the reference gene EF-1α (Escaray et al., 2014). For comparative purposes, average threshold cycle values of the control samples were used as reference.

The statistical analysis of relative gene expression was performed using the InfoStat/L program (Di Rienzo et al., 2008).

## Results

### Sequencing and read assembly

**Figure 1:**
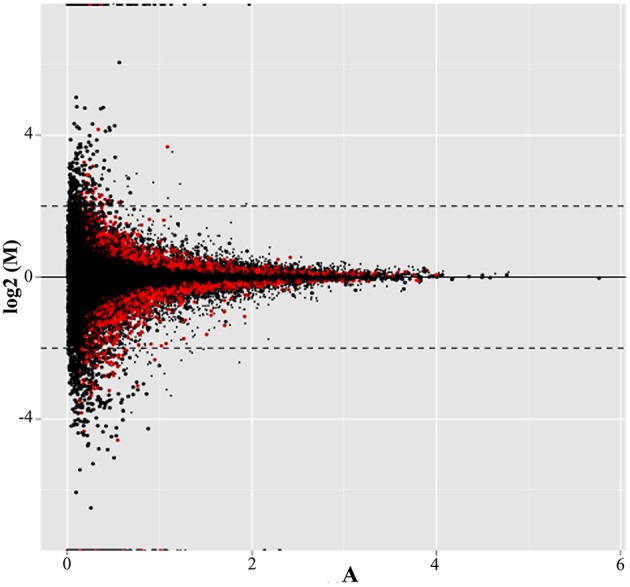
**MA-plot showing the distribution of differentially expressed genes**. The y-axis shows the log_2_-transformed FC of expression between cold stress treatment and the control. The x-axis, meanwhile, corresponds to the log_2_-transformed average expression level for each gene across all samples (FPKM). Points in red represent those genes for which *p* < 0.01. Dashed lines correspond to log_2_FC ≥ |2|.

Almost 68% of the transcripts resulting from read alignment mapped with the reference genome, allowing for the identification of 40993 genes (Figure 1). In total, 9781, or approximately 24%, were differentially expressed between treatments (*p* < 0.01, represented as red points in Figure 1). Of those genes, 1077, or about 11%, showed more than a two-fold change in their expression ratio. Within this group, 713 genes were up-regulated under cold stress treatment, while 364 were down-regulated; 133 and 75 of these genes, respectively, have no annotation in the *L. japonicus* genome. These 1077 genes were used for functional classification and annotation. It is worth noting that the percentage of mapped reads obtained was expected due to unavailability of complete genome information and the presence of highly repetitive sequences in the *L. japonicus* genome (Sato and Andersen, 2014).

### Functional annotation and classification

#### GO enrichment analysis

**Figure 2:**
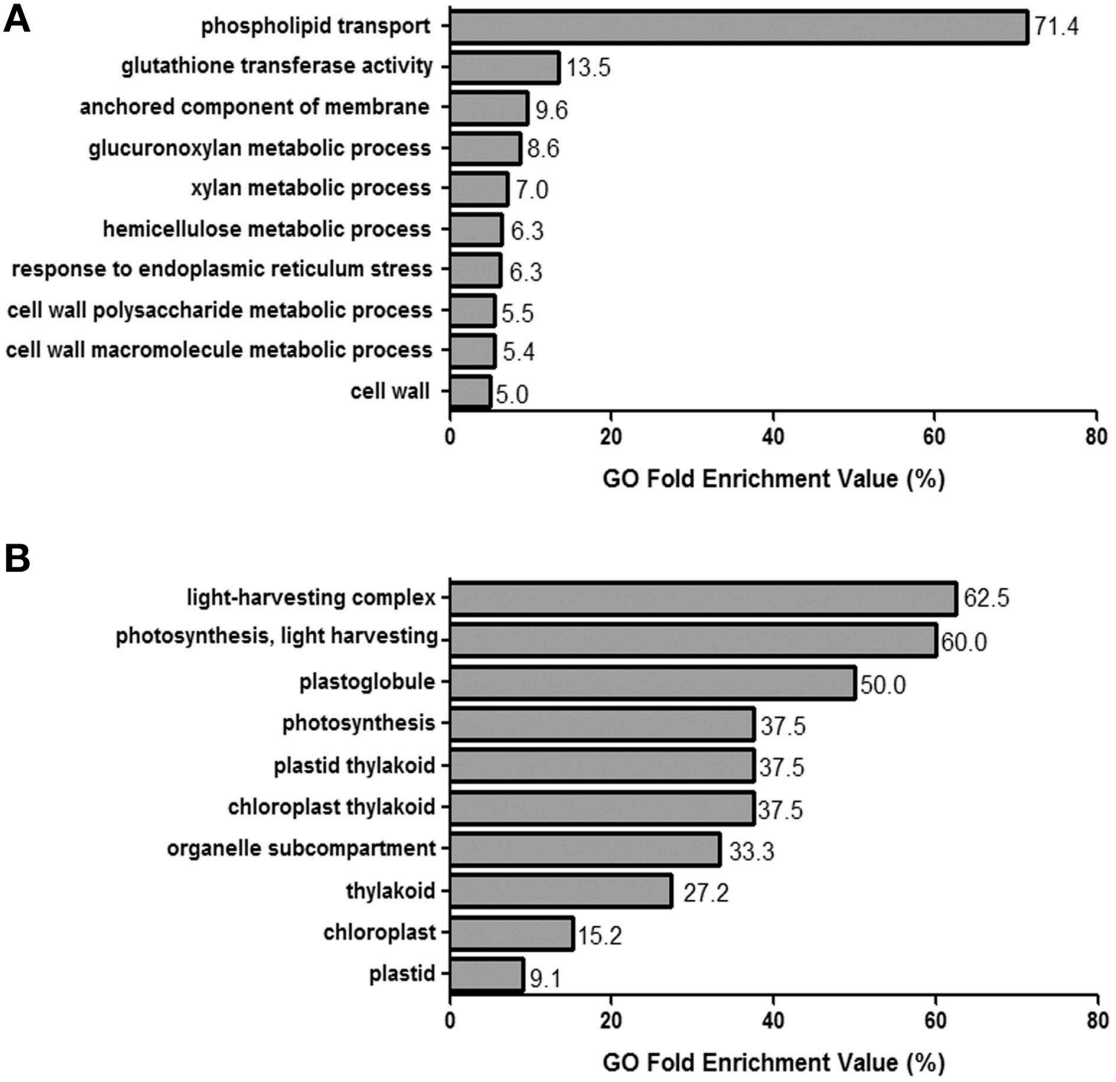
**GO enrichment analysis.(A,B) The 10 GO terms that are most representative for each set of the 713 up-regulated and 364 down-regulated genes, as defined by those for which log_2_FC≥ 2 and log_2_FC ≥ −2, respectively**. GO enrichment values (represented as percentages) were calculated by generating the ratios for the number of observed over expected GO term repeats.

GO terms were successfully identified for 87.2% of the transcripts. The ten GO terms most highly represented in the up- and down-regulated gene list, with false discovery rate *p*-values smaller than or equal to 0.01, were identified (Figures 2A,B, respectively).

The main up-regulated GO term was phospholipid transport, followed by features related to plasma membrane composition, specifically with respect to the anchored component of the membrane and response to endoplasmic reticulum stress. In addition, six of the ten up-regulated GO terms were related to the cell wall, both specifically to the cell wall and to the glucuronoxylan, xylan, hemicellulose, cell wall polysaccharide, and cell wall macromolecule metabolic processes. The gluthatione transferase activity GO term, related generally to glutathione metabolism, was also highly up-regulated. Meanwhile, the light harvesting complex, light harvesting, and photosynthesis GO terms were significantly down-regulated.

#### KEGG metabolic pathway identification

**Figure 3:**
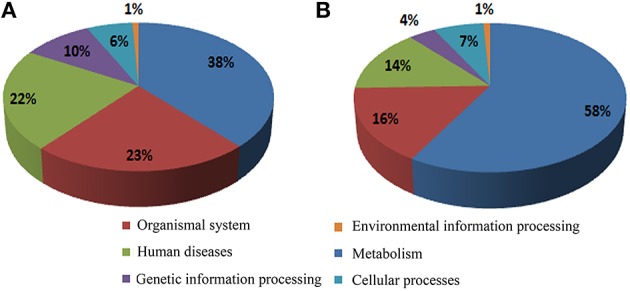
**Assignment of cold stress responsive genes to the KEGG Pathway Database. (A,B)** Classification of up- and down-regulated genes, respectively.

A total of 579 (66.6%) of the annotated genes matched a KEGG pathway, with the highest percentages pertaining to the metabolism, organismal system, and human diseases categories for both the up- and down-regulated genes (Figures 3A,B, respectively). After metabolism, organismal system was the most represented category in the KEGG analysis (Figure 3). Many of the up-regulated genes in this category were classified as heat shock protein 1/8 (K03283) and interleukin-1 receptor-associated kinase 4 [EC:2.7.11.1] (K04733) (Supplementary Table 2). Unexpectedly, the least represented category in both cases was that of environmental information processing. However, some TFs were identified in the environmental adaptation pathway (Supplementary Table 2); almost all of them were up-regulated.

**Figure 4:**
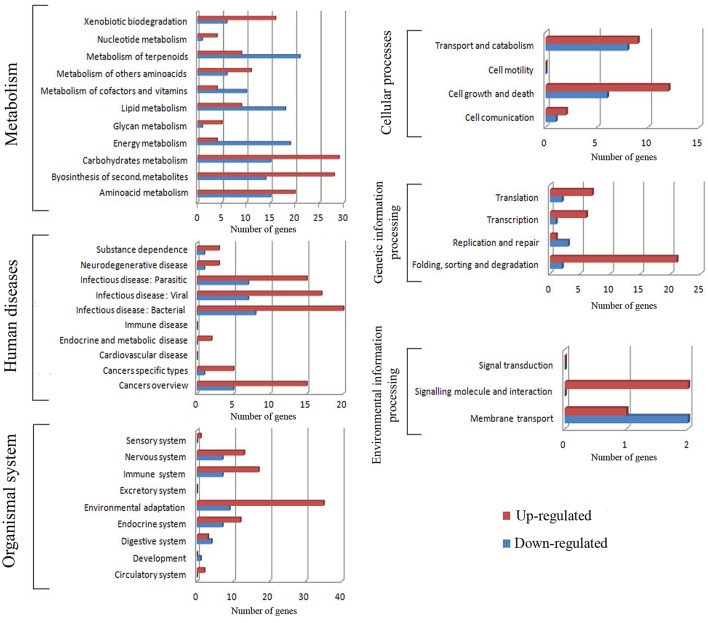
**Pathway subclassification for cold stress responsive genes for each KEGG category**.

The different genes were then further classified within these categories (Figure 4); it is worth noting that within the organismal system classification, environmental adaptation was most heavily represented, with 44 genes significantly affected. Overall, the KEGG results support conclusions drawn from the GO data. Glutathione S-transferase genes were up-regulated (Table 1), as were most of those for starch and sucrose metabolism. In addition, the transcription of some of the genes involved in arginine and proline metabolism were up-regulated by more than a factor of two. Meanwhile, light-harvesting complex proteins were down-regulated, as were the chloroplast and thylakoid membrane terms, including those of the plastoglobule, plastid thylakoid, and chloroplast thylakoid. Finally, cutin, suberine, and wax biosyntheses in the lipid metabolism category were down-regulated.

**Table 1:** **Differentially expressed genes classified under the metabolism category of the KEGG Pathway Database**.

**KEGG pathway DB metabolism**		**Gene accession**	**Log_2_FC**	***p*-value**	**KEGG annotation**	**KO**
Aminoacid metabolism	Arginine and proline metabolism	*LjSGA_056129.1*	2.10	1.00E-04	Proline dehydrogenase [EC:1.5.-.-]	K00318
		*LjSGA_006172.2*	2.23	5.00E−05	Delta-1-pyrroline-5-carboxylate synthetase [EC:2.7.2.11 1.2.1.41]	K12657
		*LjSGA_032463.1*	2.06	5.00E−05	Delta-1-pyrroline-5-carboxylate synthetase [EC:2.7.2.11 1.2.1.41]	K12657
		*LjSGA_053689.1*	2.23	5.00E−05	Delta-1-pyrroline-5-carboxylate synthetase [EC:2.7.2.11 1.2.1.41]	K12657
		*chr3.CM0711.100.r2.d*	−2.44	5.00E−05	Agmatine coumaroyltransferase [EC:2.3.1.64 2.3.1.-]	K14329
		*chr3.CM0711.120.r2.d*	−2.10	5.00E−05	Agmatine coumaroyltransferase [EC:2.3.1.64 2.3.1.-]	K14329
		*chr6.LjT40F03.150.r2.d*	−2.50	5.00E−05	Agmatine coumaroyltransferase [EC:2.3.1.64 2.3.1.-]	K14329
Byosinthesis of secondary metabolites	Anthocyanin biosynthesis	*chr4.CM0044.490.r2.m*	−2.13	5.00E−05	Anthocyanidin 3-O-glucoside 5-O-glucosyltransferase [EC:2.4.1.298]	K12338
		*chr6.CM0314.450.r2.m*	−2.50	5.00E−05	Anthocyanidin 3-O-glucoside 2”'-O-xylosyltransferase [EC:2.4.2.51]	K17193
		*LjSGA_028050.1*	−2.31	5.00E−05	Anthocyanidin 3-O-glucoside 2”'-O-xylosyltransferase [EC:2.4.2.51]	K17193
	Flavonoid biosynthesis	*chr2.CM0191.680.r2.m*	2.11	5.00E−05	Flavonol 3-O-methyltransferase [EC:2.1.1.76]	K05279
		*CM1092.70.r2.m*	2.35	5.00E−05	Polyketide reductase	K08243
		*chr2.CM0124.1170.r2.d*	4.77	5.00E−05	Leucoanthocyanidin dioxygenase [EC:1.14.11.19]	K05277
		*chr2.CM0018.1130.r2.m*	2.83	5.00E−05	Chalcone synthase [EC:2.3.1.74]	K00660
		*chr5.CM0180.660.r2.m*	2.51	7.00E−04	Chalcone isomerase [EC:5.5.1.6]	K01859
		*chr5.CM0180.670.r2.m*	2.01	5.00E−05	Chalcone isomerase [EC:5.5.1.6]	K01859
		*chr4.CM0333.580.r2.m*	2.42	5.00E−05	Flavonoid 3'-monooxygenase [EC:1.14.13.21]	K05280
		*chr3.LjT40P18.80.r2.m*	5.82	5.00E−05	Shikimate O-hydroxycinnamoyltransferase [EC:2.3.1.133]	K13065
		*chr5.CM0148.40.r2.m*	3.33	5.00E−05	Shikimate O-hydroxycinnamoyltransferase [EC:2.3.1.133]	K13065
		*chr2.CM0002.140.r2.m*	−2.27	5.00E−05	Flavonol synthase [EC:1.14.11.23]	K05278
		*chr3.CM0590.840.r2.m*	−2.71	5.00E−05	Chalcone synthase [EC:2.3.1.74]	K00660
		*LjSGA_035363.1*	−2.47	1.00E−04	Coumaroylquinate(coumaroylshikimate) 3'-monooxygenase [EC:1.14.13.36]	K09754
	Phenylpropanoid biosynthesis	*chr1.CM0221.250.r2.d*	5.13	5.00E−05	Peroxidase [EC:1.11.1.7]	K00430
		*chr2.CM1032.300.r2.m*	2.79	5.00E−05	Peroxidase [EC:1.11.1.7]	K00430
		*chr2.CM0056.580.r2.d*	2.39	5.00E−05	Beta-glucosidase [EC:3.2.1.21	K01188
		*chr6.CM0118.420.r2.m*	2.68	5.00E−05	Beta-glucosidase [EC:3.2.1.21]	K01188
		*chr6.CM0314.280.r2.m*	2.16	6.85E−03	Peroxidase [EC:1.11.1.7]	K00430
		*chr6.CM0314.270.r2.m*	2.04	5.00E−05	Peroxidase [EC:1.11.1.7]	K00430
		*LjSGA_014865.1*	2.93	2.00E−04	Peroxidase [EC:1.11.1.7]	K00430
		*LjSGA_027137.1*	2.63	1.33E−02	Peroxidase [EC:1.11.1.7]	K00430
		*LjSGA_036806.1*	2.31	5.00E−05	Peroxidase [EC:1.11.1.7]	K00430
		*LjSGA_043353.1*	2.85	5.00E−05	Peroxidase [EC:1.11.1.7]	K00430
		*LjSGA_133676.2.1*	2.27	5.00E−05	Peroxidase [EC:1.11.1.7]	K00430
		*chr3.CM0241.540.r2.d*	−2.82	5.00E−05	Ferulate-5-hydroxylase [EC:1.14.-.-]	K09755
		*chr4.CM0046.1730.r2.m*	−2.28	5.00E−05	Beta-glucosidase [EC:3.2.1.21]	K01188
		*chr5.CM0077.770.r2.d*	−2.77	5.00E−05	Peroxidase [EC:1.11.1.7]	K00430
		*chr5.CM1324.140.r2.d*	−3.33	5.00E−05	Beta-glucosidase [EC:3.2.1.21]	K01188
		*LjSGA_051150.1*	−2.26	5.00E−05	Beta-glucosidase [EC:3.2.1.21]	K01188
Carbohydrate metabolism	Starch and sucrose metabolism	*LjT44L11.110.r2.d*	3.52	4.00E−04	Pectinesterase [EC:3.1.1.11]	K01051
		*chr1.CM0122.2580.r2.m*	3.45	5.00E−05	Pectinesterase [EC:3.1.1.11]	K01051
		*chr2.CM0191.440.r2.m*	2.28	8.50E−04	Galacturan 1.4-alpha-galacturonidase [EC:3.2.1.67]	K01213
		*LjSGA_080180.1*	2.12	1.52E−02	Pectinesterase [EC:3.1.1.11]	K01051
		*chr5.CM0200.2510.r2.m*	2.23	1.08E−02	Beta-D-xylosidase 4 [EC:3.2.1.37]	K15920
		*LjSGA_148669.1*	2.74	1.65E−03	Alpha-1.4-galacturonosyltransferase [EC:2.4.1.43]	K13648
		*chr1.CM0122.2540.r2.m*	2.11	5.00E−05	Sucrose synthase [EC:2.4.1.13]	K00695
		*chr2.CM0021.1140.r2.a*	3.63	5.00E−05	Beta-amylase [EC:3.2.1.2]	K01177
		*chr2.CM0056.580.r2.d*	2.39	5.00E−05	Beta-glucosidase [EC:3.2.1.21]	K01188
		*chr4.CM0004.1850.r2.a*	2.04	5.00E−05	Alpha.alpha-trehalase [EC:3.2.1.28]	K01194
		*chr6.CM0118.420.r2.m*	2.68	5.00E−05	Beta-glucosidase [EC:3.2.1.21]	K01188
		*LjSGA_011684.1*	2.25	5.00E−05	Glucan 1.3-beta-glucosidase [EC:3.2.1.58]	K01210
		*LjSGA_032594.1*	2.33	5.00E−05	Glucan 1.3-beta-glucosidase [EC:3.2.1.58]	K01210
		*chr2.CM0081.1340.r2.m*	2.09	5.00E−05	Alpha-1.4-galacturonosyltransferase [EC:2.4.1.43]	K13648
		*chr3.CM0279.620.r2.m*	2.07	5.00E−05	Alpha-1.4-galacturonosyltransferase [EC:2.4.1.43]	K13648
		*LjSGA_054562.1*	−4.33	5.00E−05	Pectinesterase [EC:3.1.1.11]	K01051
		*LjSGA_044094.1*	−2.12	5.00E−05	Glucuronosyltransferase [EC:2.4.1.17]	K00699
		*chr4.CM0046.1730.r2.m*	−2.28	5.00E−05	Beta-glucosidase [EC:3.2.1.21]	K01188
		*chr5.CM1324.140.r2.d*	−3.33	5.00E−05	Beta-glucosidase [EC:3.2.1.21]	K01188
		*LjSGA_051150.1*	−2.26	5.00E−05	Beta-glucosidase [EC:3.2.1.21]	K01188
		*chr3.CM0091.1230.r2.d*	−2.16	5.00E−05	Glucose-1-phosphate adenylyltransferase [EC:2.7.7.27]	K00975
		*LjSGA_114621.1*	−2.13	5.00E−05	Alpha-1.4-galacturonosyltransferase [EC:2.4.1.43]	K13648
Energy metabolism	Photosynthesis - antenna proteins	*LjT24M21.140.r2.a*	−2.73	5.00E−05	Light-harvesting complex II chlorophyll a/b binding protein 4	K08915
		*chr6.CM0314.310.r2.m*	−2.48	5.00E−05	Light-harvesting complex II chlorophyll a/b binding protein 6	K08917
		*LjSGA_008354.1*	−2.97	5.00E−05	Light-harvesting complex I chlorophyll a/b binding protein 1	K08907
		*LjSGA_008355.1*	−2.87	5.00E−05	Light-harvesting complex I chlorophyll a/b binding protein 1	K08907
		*LjSGA_028870.1*	−2.40	5.00E−05	Light-harvesting complex I chlorophyll a/b binding protein 2	K08908
		*LjSGA_036743.1*	−2.41	5.00E−05	Light-harvesting complex I chlorophyll a/b binding protein 2	K08908
		*LjSGA_061139.1*	−4.14	5.00E−05	Light-harvesting complex II chlorophyll a/b binding protein 1	K08912
		*LjSGA_144948.1.1*	−2.60	5.00E−05	Light-harvesting complex I chlorophyll a/b binding protein 4	K08910
Lipid metabolism	Cutin. suberine and wax biosynthesis	*LjSGA_077301.1*	−2.11	2.00E−04	Fatty acyl-CoA reductase [EC:1.2.1.-]	K13356
		*chr4.CM0175.290.r2.m*	−2.19	5.00E−05	Omega-hydroxypalmitate O-feruloyl transferase [EC:2.3.1.188]	K15400
		*chr5.CM1323.260.r2.d*	−3.67	5.00E−05	Omega-hydroxypalmitate O-feruloyl transferase [EC:2.3.1.188]	K15400
		*LjSGA_038360.1*	−2.46	5.00E−05	Fatty acid omega-hydroxy dehydrogenase [EC:1.1.-.-]	K15403
		*LjSGA_125732.1*	−2.03	5.00E−05	Fatty acid omega-hydroxy dehydrogenase [EC:1.1.-.-]	K15403
	Steroid hormone biosynthesis	*chr2.CM0250.70.r2.m*	2.49	9.45E−03	Cytochrome P450. family 1. subfamily A. polypeptide 1 [EC:1.14.14.1]	K07408
		*LjT29E04.90.r2.d*	−2.81	5.00E−05	Cytochrome P450. family 1. subfamily A. polypeptide 1 [EC:1.14.14.1]	K07408
		*chr3.CM0451.700.r2.d*	−2.57	5.00E−05	Cytochrome P450. family 1. subfamily A. polypeptide 1 [EC:1.14.14.1]	K07408
		*LjSGA_044094.1*	−2.12	5.00E−05	Glucuronosyltransferase [EC:2.4.1.17]	K00699
Metabolism of terpenoids	BR biosynthesis	*LjSGA_081760.1*	−3.49	5.00E−05	Cytochrome P450. family 90. subfamily A. polypeptide 1 [EC:1.14.-.-]	K09588
		*LjSGA_021829.1*	−2.22	4.65E−03	Cytochrome P450 (3-epi-6-deoxocathasterone 23-monooxygenase) [EC:1.14.13.112]	K12637
		*LjSGA_038968.1*	−3.29	5.00E−05	Cytochrome P450 (3-epi-6-deoxocathasterone 23-monooxygenase) [EC:1.14.13.112]	K12637
		*LjSGA_044453.1*	−2.75	5.00E−05	Cytochrome P450. family 90. subfamily A. polypeptide 1 [EC:1.14.-.-]	K09588
		*LjSGA_068202.1*	−2.41	5.00E−05	cytochrome P450(3-epi-6-Deoxocathasterone 23-monooxygenase) [EC:1.14.13.112]	K12637
Xenobiotic biodegradation	Metabolism of xenobiotics by cytochrome P450	*chr2.CM0250.70.r2.m*	2.49	9.45E−03	Cytochrome P450. family 1. subfamily A. polypeptide 1 [EC:1.14.14.1]	K07408
		*chr4.CM0046.1480.r2.m*	2.69	9.70E−03	Glutathione S-transferase [EC:2.5.1.18]	K00799
		*chr4.CM0046.1530.r2.m*	2.43	5.00E−05	Glutathione S-transferase [EC:2.5.1.18]	K00799
		*chr5.CM0909.170.r2.m*	2.64	5.00E−05	Glutathione S-transferase [EC:2.5.1.18]	K00799
		*chr5.CM0909.210.r2.m*	2.74	5.00E−05	Glutathione S-transferase [EC:2.5.1.18]	K00799
		*LjSGA_018510.1*	3.90	5.00E−05	Glutathione S-transferase [EC:2.5.1.18]	K00799
		*LjSGA_030344.1*	3.60	5.00E−05	Glutathione S-transferase [EC:2.5.1.18]	K00799
		*LjSGA_055009.1*	2.02	5.00E−05	Glutathione S-transferase [EC:2.5.1.18]	K00799
		*LjT29E04.90.r2.d*	−2.81	5.00E−05	Cytochrome P450. family 1. subfamily A. polypeptide 1 [EC:1.14.14.1]	K07408
		*chr3.CM0451.700.r2.d*	−2.57	5.00E−05	Cytochrome P450. family 1. subfamily A. polypeptide 1 [EC:1.14.14.1]	K07408
		*LjSGA_044094.1*	−2.12	5.00E−05	Glucuronosyltransferase [EC:2.4.1.17]	K00699

Furthermore, the genes involved in the biosynthesis of secondary metabolites pathway in the KEGG metabolism category were over-represented; phenylpropanoid biosynthesis and flavonoid pathways were especially notable, with the majority of the genes up-regulated in the phenylpropanoid pathway being peroxidases [EC:1.11.1.7] (Table 1). In addition, one *CHS* ortholog (*chr2.CM0018.1130.r2.m*) and two *CHI* orthologs (*chr5.CM0180.660.r2.m* and *chr5.CM0180.670.r2.m*) were up-regulated. Finally, RNA-Seq demonstrated a down-regulation of genes related to brassinosteroid (BR) biosynthesis (included under metabolism of terpenoids) and steroid hormone biosynthesis (included under lipid metabolism).

#### TF identification and classification

**Figure 5:**
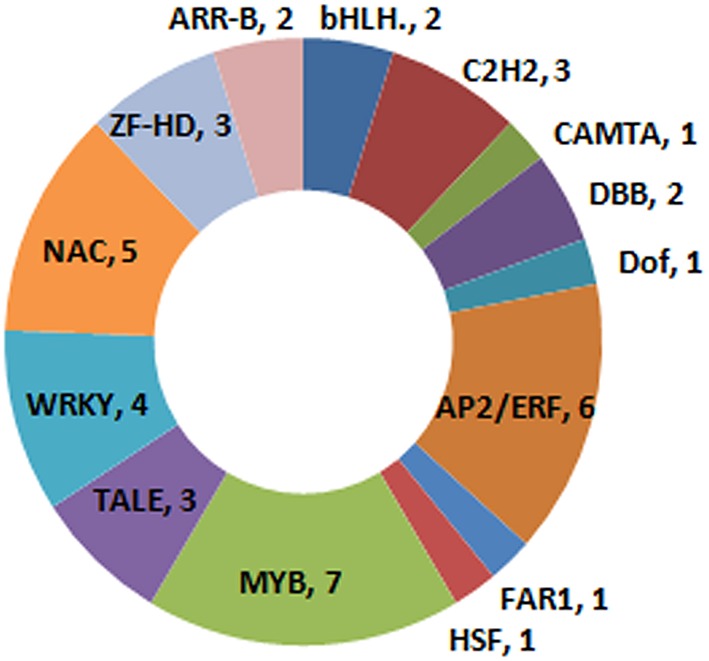
**Classification of cold stress up-regulated TFs**.

The expression levels of 41 plant TFs were up-regulated by low temperature. These TFs were sorted into 14 different families (Figure 5), with seven of these classified as MYB TFs; one of these (transcript *XLOC_019119*) was one of the two genes that had not been previously annotated (Supplementary Table 3). Additionally, five of the up-regulated TFs belonged to the NAC family, while six were part of the AP2/ERF family. One calmodulin binding transcription activator (CAMTA) TF (*chr4.CM0307.390.r2.d*) was found up-regulated, along with a number of calmodulin genes (Supplementary Tables 2, 3).

### Differentially expressed genes with no previous annotation

Overall, 50 (37.6%) and 24 (32.0%) of the up- and down- regulated genes, respectively, did not hit any sequence in the non-redundant protein GenBank database. Meanwhile, 27 (20.3%) and 20 (26.7%) of the up- and down-regulated genes, respectively, hit for an E-value greater than 10^−3^. The rest of the transcripts matched a sequence with an E-value lower than 10^−3^; however, from those, 22 (39.3%) and 10 (24.4%) of the up- and down-regulated genes, respectively, hit to an unknown protein. In all, 34 (60.7%) and 21 (75.6%) of the remaining up- and down-regulated transcripts, respectively, matched with a characterized sequence in the GenBank database (Supplementary Tables 4, 5).

Aside from the two previously discussed, non-annotated transcripts that were identified as TFs (Supplementary Table 3), other up-regulated sequences gave transcripts that hit with ribonucleases, retrotransposon proteins, serine/threonine-protein kinases, a sucrose-phosphate synthase 4, fatty acid protein elongation, and a cellulose synthase-like protein (Supplementary Table 4). Meanwhile, the down-regulated genes contained transcripts that matched with the ribonuclease H family, a serine/threonine protein kinase, a mitochondrial arginine transporter, and a nuclear pore complex protein (Supplementary Table 5).

### DREB1/CBF gene expression profiling

After 24 h of cold stress, *L. japonicus* showed no expression differences in this family of regulatory genes, though their response may be evident at shorter times. For this reason, relative expression profiles were studied.

**Figure 6:**
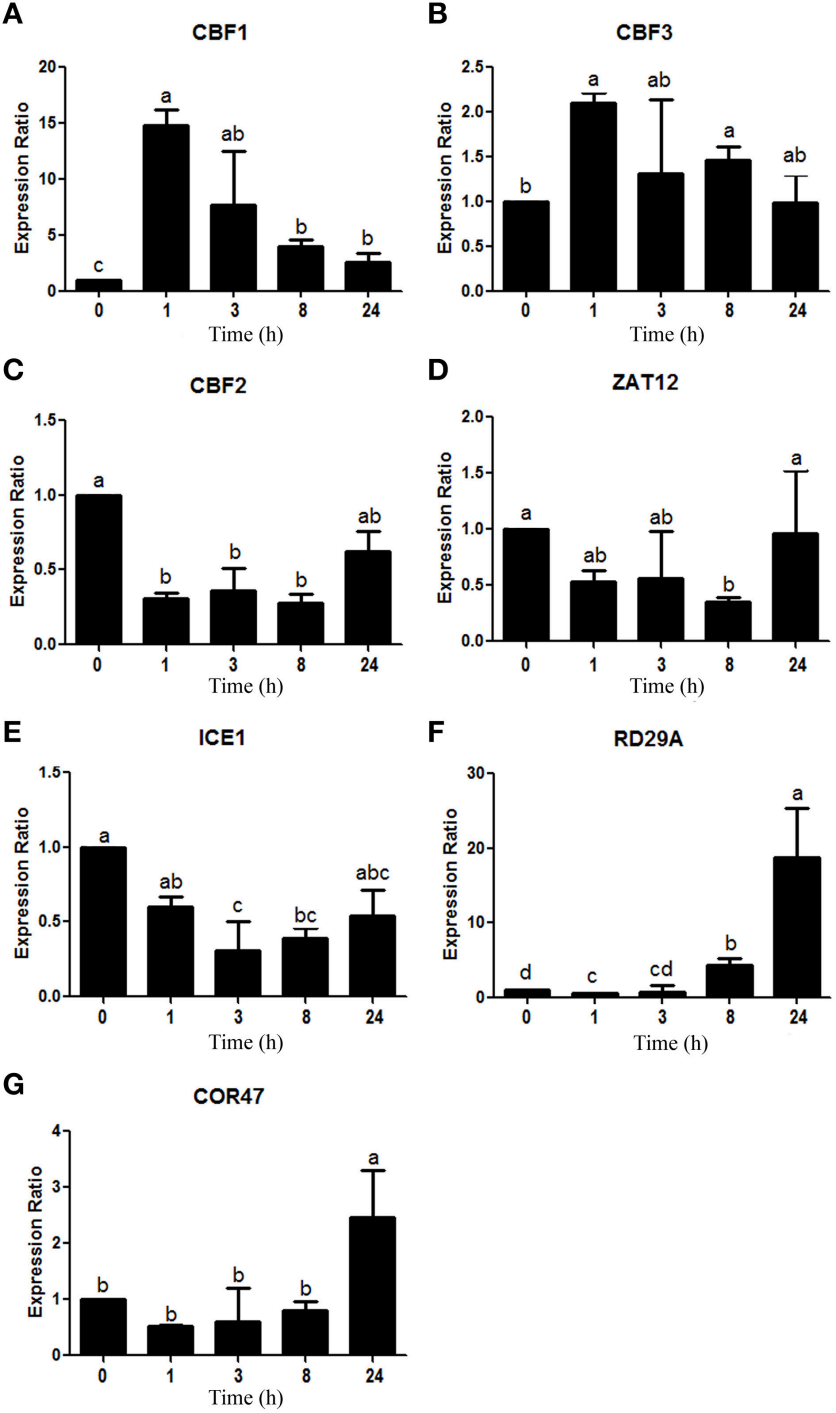
**Expression analysis of 7 genes involved in the DREB1/CBF regulon, according to qRT-PCR**. Data was obtained at 0, 1, 3, 8, and 24 h after cold stress imposition. Expression analysis of the **(A)** CBF1 (*chr4.CM0126.2110.r2.a)*, **(B)** CBF3 (*chr4.CM0126.2020.r2.a)*, **(C)** CBF2 (*chr5.CM0359.290.r2.m)*, **(D)** ZAT12 (*chr5.CM0180.280.r2.m)*, **(E)** ICE1 (*chr1.CM0104.550.r2.m)*, **(F)** RD29A (*chr5.CM0148.540.r2.m)*, and **(G)** COR47 (*chr1.CM0113.680.r2.d)* orthologs. Values represent the mean (*n* = 3) ± SEM. Means labeled with the same letters do not differ significantly (*p* < 0.05), while bars indicate standard error of the mean values.

Measurements showed a peak of CBF1 (*chr4.CM0126.2110.r2.a*) and CBF3 (*chr4.CM0126.2020.r2.a*) ortholog expression after 1 h of treatment (Figures 6A,B), though these expression levels dropped as time went on. Nonetheless, after 24 h, CBF1 expression was still significantly higher than the control conditions, even while CBF3 was no longer overexpressed. On the other hand, the relative expression pattern of the CBF2 (*chr5.CM0359.290.r2.m*) ortholog decreased after 1 h of stress and remained low throughout the treatment (Figure 6C). Meanwhile, ZAT12 (*chr5.CM0180.280.r2.m*) ortholog expression showed no significant changes (Figure 6D), even while the ICE1 (*chr1.CM0104.550.r2.m*) ortholog was down-regulated (Figure 6E). Finally, RD29A (*chr5.CM0148.540.r2.m*) and COR47 (*chr1.CM0113.680.r2.d*), two DREB1/CBF TF target genes, showed the highest expression after 8 h, although this increase in expression became evident for RD29A after just 8 h of treatment (Figures 6F,G, respectively).

## Discussion

Because metabolism was the most expressed category in the KEGG classification, we further analyzed these pathways. The organismal system category, the second most expressed, was also analyzed.

### Metabolism category

#### Lipid metabolism

The regulation results help to account for observations related to lipid metabolism in previous studies. With respect to up-regulation, earlier work had demonstrated molecular changes in the lipid membrane of plants undergoing cold and freezing stress (Iba, 2002; Welti et al., 2002) due to altered lipid biosynthesis and biomembrane rearrangement (Smolenska and Kuiper, 1977), as well as specific fatty acid changes (Willemot et al., 1977). Meanwhile, with respect to down-regulation, plants have been shown to decrease their cuticular wax content when subjected to cold stress (Shepherd and Wynne Griffiths, 2006), which is consistent not just with these results but with the negative correlation observed between frost tolerance and high epicuticular wax load in *Salix sp*. (Hietala et al., 1997).

Changes in the composition of wax due to stress must also be analyzed, as these features play a major role in stressor response; for example, two transgenic *Arabidopsis* plants with significantly enhanced drought tolerance were shown to differ in both wax composition and differential freezing response (Zhang et al., 2005, 2007).

#### Cell wall metabolism

Genes related to the cell wall are regulated by many different abiotic stresses, particularly water deficit stress and wounding; during severe low temperature conditions, extracellular ice formation could result in cell dehydration, collapse, or lysis due to ice crystal extension (Gall et al., 2015). This explains the strong up-regulation of genes involved in cell wall modification during low temperature stress, both in the literature (Domon et al., 2013; Dong et al., 2013) and in this study.

#### Xenobiotic biodegradation

Many authors have highlighted the important role of diverse gluthatione-dependent enzymes in detoxification of xenobiotics and ROS, as well as in the adaptation to different abiotic stresses, particularly cold stress (Dixon et al., 1998; Anderson and Davis, 2004; Halusková et al., 2009). During cooling, H_2_O_2_ and other ROS may accumulate, causing oxidative stress and cellular damage. In these conditions, reduced glutathione could first be oxidized by ROS, then regenerated by the activity of NADPH-dependent gluthatione reductase [EC:1.6.4.2]. Unsurprisingly, enhanced glutathione content and glutathione reductase activity have been correlated to cold stress in different plant species (Kocsy et al., 2001). In fact, overexpressing gluthatione S-transferase [EC:2.5.1.18] in transgenic rice enhances germination and growth at low temperature (Takesawa et al., 2002). Therefore, the up-regulation in glutathione transerase activity in this study comes as no surprise.

#### Photosynthesis and CO_2_ fixation

The down-regulation of terms related to photosynthesis and CO_2_ fixation are consistent with previous studies. Low temperature conditions are known to cause a reduction in maximum quantum yields for CO_2_ uptake, losses in the photochemical efficiency of photosystem II, and, with prolonged exposure to excessive light, a decreased rate of light saturated photosynthesis (Kratsch and Wise, 2000; Renaut et al., 2005). This phenomenon, photoinhibition, is associated with photodamage in plant cells and is usually used as a marker for cold tolerance (Demmig-Adams and Adams Iii, 1992; Huner et al., 1993; Long et al., 1994).

Some authors describe photosynthesis as a sensor that is capable of regulating the imbalance between energy uptake and CO_2_ fixation (Huner et al., 1993). From this perspective, photoinhibition should be the consequence of a plant's capacity to adjust photosynthetically to the prevailing environmental conditions, rather than the result of damage or injury (Huner et al., 1993; Ensminger et al., 2006). However, photodamage still should not be discounted.

Proteomics analysis of *Thellungiella halophila* at low temperature demonstrated that half of the identified cold-responsive proteins were related to chloroplast physiology and function (Gao et al., 2009), suggesting at least partial regulation of cold stress tolerance through chloroplast metabolism. A comparable analysis of rice gave similar results (Hashimoto and Komatsu, 2007), further corroborating our data.

#### Starch metabolism

Several studies have demonstrated a strong correlation between sugar concentration and cold stress tolerance (Tarkowski and Van den Ende, 2015). In fact, some key enzymes of this metabolism, specifically sucrose synthase [EC:2.4.1.13] and sucrose phosphate synthase [EC:2.4.1.14], can be regulated by low temperatures (Guy et al., 1992; Sasaki et al., 2001); however, this only applies to the first of these enzymes in this study (Table 1).

The role of starch and sucrose metabolism in this context has been reinforced in multiple mutant and transgenic plant studies. A mutant plant at the *eskimo1* (*esk1*) locus, which exhibits higher levels of soluble sugars, was frost tolerant (Xin, 1998). On the other hand, an *Arabidopsis* mutant (*sfr4*) that was deficient in tolerating freezing conditions after cold acclimation did not present cold-induced elevation of sucrose and glucose levels (Mckown et al., 1996). Meanwhile, Gilmour et al. (2000) studied the overexpression of the CBF3 TF in *Arabidopsis*, which confers cold tolerance, and found that total sugar levels were higher than those in control plants.

It could be argued that sucrose is a storage carbohydrate that can be easily catabolized when needed, making it useful either under stress or after leaving stress conditions (Pollock and Lloyd, 1987; Guy et al., 1992). Furthermore, the increase in these metabolite concentrations could be a way of maintaining carbon flux by compensating for CO_2_ assimilation and avoiding photoinhibition (Savitch et al., 2000). On the other hand, this up-regulation could also be a way to cope with osmotic impairment generated by low temperature conditions (Anchordoguy et al., 1987; Palonen and Junttila, 1999) In such cases, plants can respond through an osmotic adjustment (Xiong and Zhu, 2002; Beck et al., 2007), in a process for which sugars could be a key component (Guy et al., 1992).

#### Arginine and proline metabolism

Proline is a major organic osmolyte that accumulates in a variety of plant species in response to environmental stresses, such as drought, salinity, and extreme temperatures (Hare and Cress, 1997; Ashraf and Foolad, 2007). In plants, L-proline is synthesized from L-glutamic acid via Δ^1^-pyrroline-5-carboxylate (P5C) through P5C synthetase (P5CS) and P5C reductase. Some authors have described a transcriptional regulation of the P5CS gene, the rate-limiting factor in proline biosynthesis, under osmotic stress (Yoshiba et al., 1995, 1997).

Yoshiba et al. (1995) did not find any induction of P5CS during cold stress of *Arabidopsis thaliana*. However, this contrasts with the up-regulation of this gene observed in this study, combined with an increase in proline content in different *L. japonicus* accessions after 7 days of cold stress (data not shown). Differences in P5CS gene transcriptional regulation are to be expected, due to differences in plant species, genotype, timing, and intensity of stress imposition.

Although the actual roles of proline in plant osmotolerance remain controversial, there is supporting evidence for a positive effect on enzyme and membrane integrity, osmotic adjustment, and free radical scavenging (Kishor et al., 2005). Still, some authors have argued that proline accumulation under stress is a product of, and not an adaptive response to stress (De Lacerda et al., 2003; Maiale et al., 2004). This uncertainty calls for further study of this feature, at least in the case of *L. japonicus*.

#### Biosynthesis of secondary metabolites

Most phenolic compounds in plants are derived from the phenylpropanoid pathway and have many different physiological roles. Increases in phenolic compound content under abiotic stress, particularly with respect to flavonoids, have been extensively described (Christie et al., 1994; Dixon and Paiva, 1995), making our results consistent with previous observations.

Among the enzymes involved in the phenylpropanoid biosynthesis pathway, phenylalanine ammonia-lyase [EC:4.3.1.24] is one of the most relevant (Rivero et al., 2001). This enzyme catalyzes the transformation of L-phenylalanine into trans-cinnamic acid, and has been shown to increase in activity in response to thermal stress (Leyva et al., 1995). However, we did not find any transcriptional regulation of this particular enzyme. Nevertheless, post-transcriptional regulation is possible.

Meanwhile, phenols are oxidized by peroxidases, which increase in activity in response to different types of stress (Jansen et al., 2001; Michalak, 2006). Most flavonoids outperform well-known antioxidants, such as ascorbate, but different flavonoid oxidation processes could involve ROS scavenging as well (Hernández et al., 2009). Thus, peroxidase activity may play an essential role in these phenomena (Yamasaki et al., 1997).

Chalcone synthase [EC:2.3.1.74] and chalcone isomerase [EC:5.5.1.6] are codified by the *CHS* and *CHI* genes, respectively, and are key components of the flavonoid biosynthetic process; as such, their transcription is enhanced under cold stress (Wu et al., 2014), as was observed in this case.

#### BR biosynthesis and steroid hormone biosynthesis

BRs are steroidal plant hormones implicated in the promotion of plant growth and development, as well as in the stress response (Sasse, 1997). In fact, evidence suggests that BRs have a protecting effect at low temperatures, in that they activate cold-stress related genes COR47 and COR78 (Müssig et al., 2002; Janeczko et al., 2007; Bajguz and Hayat, 2009). However, recent studies corroborate this one in suggesting that BR biosynthesis is down-regulated at low temperatures in certain plants (Gray and Heath, 2005; Hannah et al., 2005; Kaplan et al., 2007).

This response, seemingly the opposite of what would be beneficial, was reversed by Kim et al. (2010), who studied *Arabidopsis* with a BR-insensitive 1 (*bri1*) mutation. These plants suffered from defective BR signaling, which seemingly attributed to improve tolerance to cold stress.

The role of BR in the abiotic stress response is still not clear, but our results support a possible physiological relevance of this down-regulation.

### Organismal system category

Heat-shock proteins are molecular chaperones that cope with stress-induced denaturation of other proteins (Feder and Hofmann, 1999; Sun et al., 2002; Wang et al., 2004). Indeed, Sabehat et al. (1998) showed that an induced expression of two heat-shock proteins in *Lycopersicon esculentum* L. correlated with protection against some of the symptoms of chilling injuries.

Meanwhile, interleukin-1 receptor-associated kinase 4 [EC:2.7.11.1] (K04733) is a central part of the immune and inflammatory response in mammals (Janssens and Beyaert, 2003). The signaling domains of this kinase share significant homology with some of the plant proteins that mediate activation of mitogen-activated protein kinase (MAPK) pathways (Neill and Greene, 1998). These plant proteins have been related to pathogen attack response (Dardick and Ronald, 2006), as well as abiotic stress tolerance (Lehti-Shiu et al., 2009; Rodriguez et al., 2010). Particularly, Zou et al. (2006) showed an increased expression of a receptor-associated kinase of maize (*ZmPti1*) at low temperatures. In our study, the up-regulation of some of the aforementioned genes may reflect the induction of many mitogen-activated protein kinase signaling pathways as a consequence of cold stress imposition. However, the specific gene functions remain to be further studied.

### Up-regulated TFs

Genes that are members of the AP2/ERF family are plant-specific TFs that share a conserved DNA-binding domain and participate in the abiotic stress response via specific binding to DRE/CRT, a *cis*-acting element in the promoter's target genes (Mizoi et al., 2012). AP2/ERF can be divided into four different subfamilies, namely AP2, RAV, ERF, and DREB (Mizoi et al., 2012).

Some of the most described AP2/ERF TFs in the cold stress response network belong to the DREB1/CBF (A-1) subgroup (Yamaguchi-Shinozaki and Shinozaki, 2005; Nakashima and Yamaguchi-Shinozaki, 2006). Among these, CBF3, CBF1, and CBF2 are highly similar in amino acid sequence and are quickly and transiently induced by cold stress. Their expression is ABA independent and their products activate multiple stress-inducible target genes (Mizoi et al., 2012). Interestingly, none of the AP2/ERF TFs that are up-regulated were DREB1/CBFs (Supplementary Table 3). Different authors have described characteristic expression profiles of these TFs within the first 8 h of stress (Novillo et al., 2004; Vogel et al., 2005), indicating that their expression should be studied at shorter intervals. Expression levels of some of these measured genes are analyzed in this way in the following section.

On the other hand, many studies demonstrate participation of the MYB TFs in different cold stress response pathways (Chinnusamy et al., 2007). Particularly, some are involved in the regulation of CBF TFs through the ABA independent signaling pathway (Chinnusamy et al., 2007). In fact, *MYB15* negatively regulates the expression of the CBFs, as has been shown in a mutant and transgenic *Arabidopsis* with impaired *MYB15* expression (Agarwal et al., 2006).

Some ABA-inducible MYB proteins may participate cooperatively in the ABA-dependent expression of different cold regulated genes as well (Shinozaki and Yamaguchi-shinozaki, 2000; Zhu et al., 2005; Dai et al., 2007). Because these MYB proteins are synthesized after endogenous levels of ABA accumulate, they do not play a signaling role until the later stages of the stress response. As a consequence, MYB TFs may participate in both the short and long term response to cold stress, and in an ABA-independent and/or dependent pathway.

Most of the other up-regulated TF families, such as WRKY, NAC, C2H2, and HSF, participate in different abiotic stresses responses (Chinnusamy et al., 2007; Chen et al., 2012; Scharf et al., 2012). Particularly, the NAC family may contribute to ABA-dependent gene expression under various stresses, including cold (Tran et al., 2004; Yamaguchi-Shinozaki and Shinozaki, 2005; Nakashima et al., 2007, 2012).

Meanwhile, the calcium-calmodulin-CAMTA complex participates in the transcription regulation of different target genes. In this sense, cellular calcium(II) levels act as a secondary messenger that modulates diverse physiological processes that are important for stress adaptation. In particular, a rapid calcium influx is required for proper cold acclimation (Doherty et al., 2009).

In addition, a possible function of the calcium-calmodulin-CAMTA complex was suggested in the DREB1/CBF signaling pathway (Thomashow, 2010). In fact, Doherty et al. (2009) proved that CBF1, CBF2, and ZAT12 were direct targets of CAMTA3 (Doherty et al., 2009). The evidence that CAMTA TFs and calcium signaling are upstream of the DREB1/CBF regulon make them relevant in the first few hours of stress imposition. However, this does not mean that calcium signaling does not play a role at longer timeframes. Thus, an induction in *chr4.CM0307.390.r2.d* gene expression may imply that calcium participates in the cold stress response in a DREB1/CBF independent pathway or downstream from their target genes, a hypothesis that is reinforced by our calmodulin gene expression data.

### The DREB1/CBF response

The DREB1/CBF regulatory genes are an AP2/ERF TF subgroup that binds to the DRE/CRT regulatory element present in the promoters of target genes (Yamaguchi-Shinozaki and Shinozaki, 2005; Mizoi et al., 2012). These genes have been well characterized as key components of the ABA-independent cold stress response, mainly in *Arabidopsis* (Thomashow, 2010; Medina et al., 2011). In fact, a distinctive CBF pattern expression has been described for within the first 8 h (Novillo et al., 2004; Vogel et al., 2005). Remarkably, until now, no CBF expression studies have been performed for the *Lotus* genus.

The CBF1 and CBF3 results of this study are in agreement with those obtained by Novillo et al. (2004) and Cook et al. (2004), which showed maximum expression levels within the first 2 h of cold stress in *Arabidopsis* plants. Different authors have also described a similar expression pattern for CBF2, reaching a maximum only several hours later than CBF1 and CBF3 (Cook et al., 2004; Novillo et al., 2004, 2007). The observed differences in our study may be explained by the fact that most pertinent studies were completed using *Arabidopsis*, with only a few using legume species like *Medicago* spp. (Pennycooke et al., 2008; Zhang et al., 2011). In fact, there is no conclusive evidence for this expression pattern in *L. japonicus*. Interestingly, CBF2 has been described as a negative regulator of CBF1 and CBF3 (Novillo et al., 2004; Medina et al., 2011). As a consequence, the initial reduction observed in its expression level in *L. japonicus* might actually support the increase in CBF1 and CBF3.

Like CBF2, ZAT12 has been described as a negative regulator of DREB1/CBFs, though it is generally induced in this timeframe in *Arabidopsis* (Vogel et al., 2005; Zhai et al., 2010). However, there is no evidence of this TF behavior in legumes.

On the other hand, one TF that positively regulates DREB1/CBF TF expression is the MYC-like bHLH protein ICE1 (Yamaguchi-Shinozaki and Shinozaki, 2006). The relevance of this TF in cold stress response has been demonstrated by *ice1 Arabidopsis* mutants in which DREB1/CBFs expression was deregulated, leading to a reduced expression of many of their downstream cold-responsive genes (Chinnusamy et al., 2003; Lee et al., 2005). However, it was shown that a cold induced post-translational modification of ICE1, rather than an increase in expression, allowed for the positive regulation of the DREB1/CBF regulon (Sharma et al., 2005). Further studies should be carried out to clarify the role of ICE1 in the cold stress response of *L. japonicus*.

Finally, the expression patterns of RD29A and COR47 are consistent with the fact that these are known *cor* genes that drive the cold stress response in plants several hours after stress conditions begin (Thomashow et al., 2001).

## Conclusion

Our study provides novel insights into the molecular mechanisms of *L. japonicus* cold stress response.Overall, 1077 differentially expressed genes were obtained using RNA-Seq data, consisting of 713 that were up-regulated and 364 that were down-regulated. These genes were classified by their functional annotation through BLASTx, GO, and KEGG analysis.

Cold stress in *L. japonicus* modulates gene expression of lipid and cell wall metabolism, together with transcription regulation of genes related to flavonoid and phenylpropanoid biosynthesis. Genes related to xenobiotic degradation, heat-shock proteins, and starch and proline metabolism were up-regulated. In contrast, photosynthesis and chloroplast related genes were down-regulated, demonstrating a dependency of cold acclimation on the photosynthetic process.

Different types of TFs were up-regulated as well, with the MYB, AP2/ERF, and NAC families being the most numerous. Interestingly, two putative novel TFs were identified that participate in cold response. Finally, the expression profiles of some DREB1/CBFs and *cor* genes were described for the first 8 h of stress. To our knowledge, this is the first report of DREB1/CBF expression under low temperature conditions in this genus.

Our results constitute the first transcriptome profiling of the model legume *L. japonicus* under cold stress. Data obtained allowed for the identification of possible target metabolisms, information that could be used to improve stress tolerance in *L. japonicus*, not to mention other members of the genus, in the future.

## Author contributions

PC, SM, OR, and FE conceived and designed the experiments. PC performed the experiments. PC and FE analyzed the data. OR contributed reagents, materials, and analytical tools. PC, SM, OR, and FE wrote the paper. All authors read and approved the final manuscript.

## Funding

CONICET (PIP 0980). ANPCYT (PICT 1612)-PhD Oscar Adolfo Ruiz.

## Author information

PC is a CONICET fellow. SM, OR, and FE are career research members of CONICET.

### Conflict of interest statement

The authors declare that the research was conducted in the absence of any commercial or financial relationships that could be construed as a potential conflict of interest.
