# Supplementary material for: Transcriptome Response Mediated by Cold Stress in Lotus japonicus
Source: Front Plant Sci. 2016 Mar 30;7:374. doi: 10.3389/fpls.2016.00374 (PMC4811897; doi:10.3389/fpls.2016.00374)
Supplement: Supplementary file 1 [file Table1.DOCX]

Supplementary Material

Transcriptome response mediated by cold stress in Lotus japonicus

Pablo Ignacio Calzadilla, Santiago Javier Maiale, Oscar Adolfo^*^ Ruiz and Francisco José Escaray.

*** Correspondence:** ruiz@intech.gov.ar

## Supplementary Table 1. Primers used for qRT-PCR.

| **Gene ID** | **Forward primer** | **Reverse primer** |
| --- | --- | --- |
| *chr1.CM0104.550.r2.m* | GTTGGACAACCTCGGATTGG | CTGGCCTTCTGTGCATTGCT |
| *chr3.CM0091.1980.r2.m* | GCATTCTGGCCATTTTGGA | GCCCACTTGCTCAAGCTTCT |
| *chr1.CM0113.680.r2.d* | CACCGATCTGACAGCAGCTCTA | CATCTGTACTCTTCACCTCTTTCTTTTC |
| *chr5.CM0180.280.r2.m* | CAGCAGGGAGGTCCATATGAA | GGAACTTCCGGTTGCATGTC |
| *chr5.CM0148.540.r2.m* | GATGTTTCTGACCCCTCAGGAT | CCTCTGCTACCTTCCCATAAACTG |
| *chr4.CM0126.2020.r2.a* | CGGAAGCAGGTTCCTCTAGCT | CGGTACACAGGGTGCCTTGT |
| *chr4.CM0126.2110.r2.a* | GAAGTCCGCTTGCCTCAACT | TTCCGTTGCCACTCTCCTAAG |
| *chr5.CM0359.290.r2.m* | TGAAACGCGACACCCTACCT | GAGCCACACACGCGCTTT |
| *LjSGA_021886.2* | TGAGCTTGTGAAGGTTGG | AACAGGGAGTTGACAAATCT |
| *chr1.CM0378.230.r2.m* | AAGATGGAGAGGGATATGG | GTCTTGTTCTCACGCTTT |
| *LjSGA_063085.1* | ATACAACTACAGCGTCAT | GCAATCAATTTGGACTCA |
