# Supplementary material for: Transcriptome Response Mediated by Cold Stress in Lotus japonicus
Source: Front Plant Sci. 2016 Mar 30;7:374. doi: 10.3389/fpls.2016.00374 (PMC4811897; doi:10.3389/fpls.2016.00374)
Supplement: Supplementary file 2 [file Table2.DOCX]

Supplementary Material

**Transcriptome response mediated by cold stress in *Lotus japonicus***

Pablo Ignacio Calzadilla, Santiago Javier Maiale, Oscar Adolfo^*^ Ruiz and Francisco José Escaray.

*** Correspondence:** ruiz@intech.gov.ar

**Supplementary Table 2.** **Differentially expressed genes classified under the organismal system category of the KEGG Pathway Database.**

| **Kegg Pathways DB** |  | **Gene ID** | **Log_2_ FC** | **p value** | **Functional annotation** | **Kegg ID** |
| --- | --- | --- | --- | --- | --- | --- |
| **Organismal system** | **Endocryne system** | *chr1.CM0637.320.r2.a* | 2.31 | 1.50E-04 | heat shock 70kDa protein 1/8 | [K03283](http://www.genome.jp/dbget-bin/www_bget?ko:K03283) |
|  |  | *chr3.CM0786.370.r2.a* | 3.74 | 5.00E-05 | heat shock 70kDa protein 1/8 | [K03283](http://www.genome.jp/dbget-bin/www_bget?ko:K03283) |
|  |  | *chr4.CM0179.510.r2.d* | 3.94 | 5.00E-05 | heat shock 70kDa protein 1/8 | [K03283](http://www.genome.jp/dbget-bin/www_bget?ko:K03283) |
|  |  | *LjSGA_034228.1* | 3.83 | 5.00E-05 | heat shock 70kDa protein 1/8 | [K03283](http://www.genome.jp/dbget-bin/www_bget?ko:K03283) |
|  |  | *LjSGA_076792.1* | 4.05 | 5.00E-05 | heat shock 70kDa protein 1/8 | [K03283](http://www.genome.jp/dbget-bin/www_bget?ko:K03283) |
|  |  | *LjSGA_015689.1* | -2.68 | 5.00E-05 | heat shock 70kDa protein 1/8 | [K03283](http://www.genome.jp/dbget-bin/www_bget?ko:K03283) |
|  | **Environmental adaptation** | *chr1.CM0121.30.r2.m* | 2.91 | 5.00E-05 | calmodulin | [K02183](http://www.genome.jp/dbget-bin/www_bget?ko:K02183) |
|  |  | *LjT47J13.70.r2.a* | 2.68 | 5.00E-05 | zinc finger protein CONSTANS | [K12135](http://www.genome.jp/dbget-bin/www_bget?ko:K12135) |
|  |  | *chr1.CM1868.80.r2.a* | 4.16 | 5.00E-05 | Dof zinc finger protein DOF5.5 | [K16222](http://www.genome.jp/dbget-bin/www_bget?ko:K16222) |
|  |  | *chr1.CM0122.1220.r2.m* | 2.34 | 5.00E-05 | phytochrome-interacting factor 3 | [K12126](http://www.genome.jp/dbget-bin/www_bget?ko:K12126) |
|  |  | *chr3.CM0155.170.r2.d* | 3.17 | 5.00E-05 | zinc finger protein CONSTANS | [K12135](http://www.genome.jp/dbget-bin/www_bget?ko:K12135) |
|  |  | *chr4.CM0087.500.r2.m* | 3.18 | 5.00E-05 | pseudo-response regulator 1 | [K12127](http://www.genome.jp/dbget-bin/www_bget?ko:K12127) |
|  |  | *chr4.CM0042.1360.r2.m* | 5.08 | 5.00E-05 | flavin-binding kelch repeat F-box protein 1 | [K12116](http://www.genome.jp/dbget-bin/www_bget?ko:K12116) |
|  |  | *chr5.CM0456.540.r2.m* | 4.10 | 5.00E-05 | MYB-related TF LHY | [K12133](http://www.genome.jp/dbget-bin/www_bget?ko:K12133) |
|  |  | *LjSGA_092681.1* | 3.99 | 5.00E-05 | pseudo-response regulator 5 | [K12130](http://www.genome.jp/dbget-bin/www_bget?ko:K12130) |
|  |  | *chr4.CM0042.2080.r2.m* | -4.03 | 5.00E-05 | protein FLOWERING LOCUS T | [K16223](http://www.genome.jp/dbget-bin/www_bget?ko:K16223) |
|  |  | *LjSGA_100666.1* | -3.14 | 5.00E-05 | zinc finger protein CONSTANS | [K12135](http://www.genome.jp/dbget-bin/www_bget?ko:K12135) |
|  |  | *chr3.CM0106.190.r2.m* | 2.45 | 5.00E-05 | calcium-binding protein CML | [K13448](http://www.genome.jp/dbget-bin/www_bget?ko:K13448) |
|  |  | *LjT06N06.390.r2.d* | 2.78 | 5.00E-05 | brassinosteroid insensitive 1-associated receptor kinase 1 [EC:2.7.10.1 2.7.11.1] | [K13416](http://www.genome.jp/dbget-bin/www_bget?ko:K13416) |
|  |  | *chr1.CM0104.1860.r2.d* | 2.30 | 5.00E-05 | disease resistance protein RPS4 | [K16226](http://www.genome.jp/dbget-bin/www_bget?ko:K16226) |
|  |  | *chr1.CM0393.520.r2.d* | 3.07 | 5.00E-05 | disease resistance protein RPS4 | [K16226](http://www.genome.jp/dbget-bin/www_bget?ko:K16226) |
|  |  | *chr1.CM0105.740.r2.a* | 3.38 | 5.00E-05 | probable WRKY TF 52 | [K16225](http://www.genome.jp/dbget-bin/www_bget?ko:K16225) |
|  |  | *chr2.LjB15M17.60.r2.m* | 3.04 | 5.00E-05 | LRR receptor-like serine/threonine-protein kinase FLS2 [EC:2.7.11.1] | [K13420](http://www.genome.jp/dbget-bin/www_bget?ko:K13420) |
|  |  | *chr2.CM0020.160.r2.d* | 2.26 | 5.00E-05 | disease resistance protein RPM1 | [K13457](http://www.genome.jp/dbget-bin/www_bget?ko:K13457) |
|  |  | *chr4.CM0161.190.r2.d* | 2.81 | 5.50E-04 | brassinosteroid insensitive 1-associated receptor kinase 1 [EC:2.7.10.1 2.7.11.1] | [K13416](http://www.genome.jp/dbget-bin/www_bget?ko:K13416) |
|  |  | *chr4.CM1622.200.r2.a* | 2.76 | 5.00E-05 | WRKY TF 33 | [K13424](http://www.genome.jp/dbget-bin/www_bget?ko:K13424) |
|  |  | *chr5.CM0148.470.r2.m* | 2.26 | 5.00E-05 | LRR receptor-like serine/threonine-protein kinase FLS2 [EC:2.7.11.1] | [K13420](http://www.genome.jp/dbget-bin/www_bget?ko:K13420) |
|  |  | *chr5.CM0456.520.r2.m* | 2.31 | 5.00E-05 | LRR receptor-like serine/threonine-protein kinase FLS2 [EC:2.7.11.1] | [K13420](http://www.genome.jp/dbget-bin/www_bget?ko:K13420) |
|  |  | *chr6.CM0679.520.r2.d* | 2.63 | 5.00E-05 | disease resistance protein RPS4 | [K16226](http://www.genome.jp/dbget-bin/www_bget?ko:K16226) |
|  |  | *LjSGA_012799.2* | 2.10 | 5.00E-05 | WRKY TF 33 | [K13424](http://www.genome.jp/dbget-bin/www_bget?ko:K13424) |
|  |  | *LjSGA_017567.1* | 2.67 | 5.00E-05 | LRR receptor-like serine/threonine-protein kinase EFR [EC:2.7.11.1] | [K13428](http://www.genome.jp/dbget-bin/www_bget?ko:K13428) |
|  |  | *LjSGA_023624.0.1* | 3.81 | 8.65E-03 | calcium-binding protein CML | [K13448](http://www.genome.jp/dbget-bin/www_bget?ko:K13448) |
|  |  | *LjSGA_033122.2* | 6.39 | 5.00E-05 | molecular chaperone HtpG | [K04079](http://www.genome.jp/dbget-bin/www_bget?ko:K04079) |
|  |  | *LjSGA_033246.1* | 6.00 | 5.00E-05 | molecular chaperone HtpG | [K04079](http://www.genome.jp/dbget-bin/www_bget?ko:K04079) |
|  |  | *LjSGA_034276.1* | 2.51 | 5.00E-05 | respiratory burst oxidase [EC:1.6.3.- 1.11.1.-] | [K13447](http://www.genome.jp/dbget-bin/www_bget?ko:K13447) |
|  |  | *LjSGA_063791.1* | 2.03 | 6.85E-03 | calcium-binding protein CML | [K13448](http://www.genome.jp/dbget-bin/www_bget?ko:K13448) |
|  |  | *LjSGA_064721.0.1* | 2.00 | 5.00E-05 | molecular chaperone HtpG | [K04079](http://www.genome.jp/dbget-bin/www_bget?ko:K04079) |
|  |  | *LjSGA_074430.1* | 2.56 | 5.00E-05 | calcium-binding protein CML | [K13448](http://www.genome.jp/dbget-bin/www_bget?ko:K13448) |
|  |  | *LjSGA_113688.1* | 2.29 | 2.70E-03 | LRR receptor-like serine/threonine-protein kinase EFR [EC:2.7.11.1] | [K13428](http://www.genome.jp/dbget-bin/www_bget?ko:K13428) |
|  |  | *chr3.CM0208.290.r2.m* | -2.15 | 5.00E-05 | TF MYC2 | [K13422](http://www.genome.jp/dbget-bin/www_bget?ko:K13422) |
|  |  | *LjSGA_061207.1* | -2.10 | 5.00E-05 | disease resistance protein RPS4 | [K16226](http://www.genome.jp/dbget-bin/www_bget?ko:K16226) |
|  |  | *LjSGA_134572.1* | -2.47 | 5.00E-05 | pathogenesis-related protein 1 | [K13449](http://www.genome.jp/dbget-bin/www_bget?ko:K13449) |
|  | **Immune system** | *chr1.CM0163.10.r2.d* | 2.15 | 1.65E-03 | interleukin-1 receptor-associated kinase 4 [EC:2.7.11.1] | [K04733](http://www.genome.jp/dbget-bin/www_bget?ko:K04733) |
|  |  | *chr2.CM0008.460.r2.m* | 2.57 | 5.00E-05 | interleukin-1 receptor-associated kinase 4 [EC:2.7.11.1] | [K04733](http://www.genome.jp/dbget-bin/www_bget?ko:K04733) |
|  |  | *chr3.CM0005.410.r2.m* | 2.01 | 5.00E-05 | interleukin-1 receptor-associated kinase 4 [EC:2.7.11.1] | [K04733](http://www.genome.jp/dbget-bin/www_bget?ko:K04733) |
|  |  | *chr4.CM0161.220.r2.d* | 2.22 | 5.00E-05 | interleukin-1 receptor-associated kinase 4 [EC:2.7.11.1] | [K04733](http://www.genome.jp/dbget-bin/www_bget?ko:K04733) |
|  |  | *chr4.CM0042.860.r2.m* | 2.97 | 5.00E-05 | interleukin-1 receptor-associated kinase 4 [EC:2.7.11.1] | [K04733](http://www.genome.jp/dbget-bin/www_bget?ko:K04733) |
|  |  | *chr5.CM0328.1020.r2.d* | 2.54 | 5.00E-05 | interleukin-1 receptor-associated kinase 4 [EC:2.7.11.1] | [K04733](http://www.genome.jp/dbget-bin/www_bget?ko:K04733) |
|  |  | *LjSGA_038530.1* | 4.10 | 6.40E-03 | interleukin-1 receptor-associated kinase 4 [EC:2.7.11.1] | [K04733](http://www.genome.jp/dbget-bin/www_bget?ko:K04733) |
|  |  | *LjSGA_086627.1* | 2.04 | 5.00E-05 | interleukin-1 receptor-associated kinase 4 [EC:2.7.11.1] | [K04733](http://www.genome.jp/dbget-bin/www_bget?ko:K04733) |
|  |  | *chr3.CM0005.420.r2.m* | -2.22 | 5.00E-05 | interleukin-1 receptor-associated kinase 4 [EC:2.7.11.1] | [K04733](http://www.genome.jp/dbget-bin/www_bget?ko:K04733) |
