# Supplementary material for: Transcriptome Response Mediated by Cold Stress in Lotus japonicus
Source: Front Plant Sci. 2016 Mar 30;7:374. doi: 10.3389/fpls.2016.00374 (PMC4811897; doi:10.3389/fpls.2016.00374)
Supplement: Supplementary file 3 [file Table3.DOCX]

Supplementary Material

**Transcriptome response mediated by cold stress in *Lotus japonicus***

Pablo Ignacio Calzadilla, Santiago Javier Maiale, Oscar Adolfo^*^ Ruiz and Francisco José Escaray.

*** Correspondence:** ruiz@intech.gov.ar

**Supplementary Table 3.** **Cold stress up-regulated TFs*.*** Sequences labeled as NA were not annotated in the KEGG Database. Putative novel TFs are underlined.

| Gene Accession | Log2 FC | p-value | TF Family | KEGG Annotation | KO | Blastx Results |
| --- | --- | --- | --- | --- | --- | --- |
| chr2.CM0060.290.r2.a | 2.69 | 5.00E-05 | AP2/ERF | EREBP-like factor | [K09286](http://www.genome.jp/dbget-bin/www_bget?ko:K09286) | [Ethylene-responsive TF ERF061](http://blast.ncbi.nlm.nih.gov/Blast.cgi#alnHdr_357481437) |
| chr4.CM0536.270.r2.d | 2.39 | 5.00E-05 | AP2/ERF | EREBP-like factor | [K09286](http://www.genome.jp/dbget-bin/www_bget?ko:K09286) | [ethylene-responsive TF ERF118-like](http://blast.ncbi.nlm.nih.gov/Blast.cgi#alnHdr_449525265) |
| chr5.CM0494.320.r2.m | 2.33 | 2.80E-03 | AP2/ERF | ethylene-responsive TF 1 | [K14516](http://www.genome.jp/dbget-bin/www_bget?ko:K14516) | [ethylene-responsive TF 1B-like](http://blast.ncbi.nlm.nih.gov/Blast.cgi#alnHdr_356545857) |
| chr5.CM0052.670.r2.d | 2.07 | 5.00E-05 | AP2/ERF | EREBP-like factor | [K09286](http://www.genome.jp/dbget-bin/www_bget?ko:K09286) | [ethylene-responsive TF ERF110-like](http://blast.ncbi.nlm.nih.gov/Blast.cgi#alnHdr_502118939) |
| LjSGA_025266.1 | 3.33 | 5.00E-05 | AP2/ERF | EREBP-like factor | [K09286](http://www.genome.jp/dbget-bin/www_bget?ko:K09286) | [ethylene-responsive TF ERF053-like](http://blast.ncbi.nlm.nih.gov/Blast.cgi#alnHdr_356547020) |
| LjSGA_034198.1 | 2.63 | 5.00E-05 | AP2/ERF | EREBP-like factor | [K09286](http://www.genome.jp/dbget-bin/www_bget?ko:K09286) | [ethylene-responsive TF ERF053-like](http://blast.ncbi.nlm.nih.gov/Blast.cgi#alnHdr_571462590) |
| chr4.CM0087.500.r2.m | 3.18 | 5.00E-05 | ARR-B/CO-Like | pseudo-response regulator 1 | K12127 | [Two-component response regulator-like APRR1](http://blast.ncbi.nlm.nih.gov/blast/Blast.cgi#alnHdr_734437696) |
| LjSGA_092681.1 | 3.99 | 5.00E-05 | ARR-B | pseudo-response regulator 5 | K12130 | [Two-component response regulator-like PRR95](http://blast.ncbi.nlm.nih.gov/blast/Blast.cgi#alnHdr_734381713) |
| chr1.CM0122.1220.r2.m | 2.34 | 5.00E-05 | bHLH | phytochrome-interacting factor 3 | [K12126](http://www.genome.jp/dbget-bin/www_bget?ko:K12126) | TF PIF3-like |
| chr4.CM0307.390.r2.d | 3.20 | 5.00E-05 | CAMTA | myosin V | [K10357](http://www.genome.jp/dbget-bin/www_bget?ko:K10357) | [Calmodulin-binding transcription activator 2](http://blast.ncbi.nlm.nih.gov/Blast.cgi#alnHdr_734329922) |
| chr2.CM0021.1970.r2.m | 2.12 | 5.00E-05 | C2H2 | KRAB domain-containing zinc finger protein | [K09228](http://www.genome.jp/dbget-bin/www_bget?ko:K09228) | [Zinc finger protein ZAT9](http://blast.ncbi.nlm.nih.gov/Blast.cgi#alnHdr_734424467) |
| LjT47J13.70.r2.a | 2.68 | 5.00E-05 | DBB | zinc finger protein CONSTANS | [K12135](http://www.genome.jp/dbget-bin/www_bget?ko:K12135) | [B-box type zinc finger protein](http://blast.ncbi.nlm.nih.gov/Blast.cgi#alnHdr_657399039) |
| chr3.CM0155.170.r2.d | 3.17 | 5.00E-05 | DBB | zinc finger protein CONSTANS | [K12135](http://www.genome.jp/dbget-bin/www_bget?ko:K12135) | Zinc finger protein CONSTANS-LIKE 14 |
| chr1.CM1868.80.r2.a | 4.16 | 5.00E-05 | Dof | Dof zinc finger protein DOF5.5 | [K16222](http://www.genome.jp/dbget-bin/www_bget?ko:K16222) | [dof zinc finger protein DOF5.4](http://blast.ncbi.nlm.nih.gov/Blast.cgi#alnHdr_225455716) |
| LjT15C06.80.r2.m | 2.19 | 5.00E-05 | FAR1 | zinc finger SWIM domain-containing protein 3 | [K17604](http://www.genome.jp/dbget-bin/www_bget?ko:K17604) | [protein FAR1-RELATED SEQUENCE 5-like](http://blast.ncbi.nlm.nih.gov/Blast.cgi#alnHdr_571552034) |
| chr1.CM0133.500.r2.m | 3.55 | 5.00E-05 | HSF | heat shock TF. other eukaryote | [K09419](http://www.genome.jp/dbget-bin/www_bget?ko:K09419) | [heat shock factor protein HSF30-like](http://blast.ncbi.nlm.nih.gov/Blast.cgi#alnHdr_566006193) |
| chr1.CM0122.1190.r2.m | 2.07 | 5.00E-05 | MYB | myb proto-oncogene protein. Plant | [K09422](http://www.genome.jp/dbget-bin/www_bget?ko:K09422) | [TF MYB86](http://blast.ncbi.nlm.nih.gov/Blast.cgi#alnHdr_734425725) |
| chr2.CM0435.1360.r2.m | 2.57 | 2.50E-04 | MYB | myb proto-oncogene protein. Plant | [K09422](http://www.genome.jp/dbget-bin/www_bget?ko:K09422) | [TF MYB44](http://blast.ncbi.nlm.nih.gov/Blast.cgi#alnHdr_734426064) |
| chr4.CM0042.700.r2.m | 2.45 | 5.00E-05 | MYB | myb proto-oncogene protein. Plant | [K09422](http://www.genome.jp/dbget-bin/www_bget?ko:K09422) | [TF MYB21](http://blast.ncbi.nlm.nih.gov/Blast.cgi#alnHdr_734363509) |
| chr2.CM0168.360.r2.d | 2.27 | 3.60E-03 | MYB_related | myb proto-oncogene protein. Plant | [K09422](http://www.genome.jp/dbget-bin/www_bget?ko:K09422) | [telomere repeat-binding factor 2-like](http://blast.ncbi.nlm.nih.gov/Blast.cgi#alnHdr_502152722) |
| chr4.CM0126.410.r2.d | 2.44 | 4.60E-03 | MYB_related | myb proto-oncogene protein. Plant | [K09422](http://www.genome.jp/dbget-bin/www_bget?ko:K09422) | [protein ODORANT1](http://blast.ncbi.nlm.nih.gov/Blast.cgi#alnHdr_356495619) |
| chr5.CM0456.540.r2.m | 4.10 | 5.00E-05 | MYB_related | MYB-related TF LHY | [K12133](http://www.genome.jp/dbget-bin/www_bget?ko:K12133) | [TF ASG4-like isoform X1](http://blast.ncbi.nlm.nih.gov/Blast.cgi#alnHdr_571484310) |
| chr6.CM0367.750.r2.m | 2.27 | 5.00E-05 | TALE | homeobox protein Meis1 | [K15613](http://www.genome.jp/dbget-bin/www_bget?ko:K15613) | [Knox class 1 protein](http://blast.ncbi.nlm.nih.gov/Blast.cgi#alnHdr_3426304) |
| LjSGA_010170.1 | 2.10 | 7.80E-03 | TALE | homeobox protein homothorax | [K16672](http://www.genome.jp/dbget-bin/www_bget?ko:K16672) | [homeobox knotted-1-like protein KNOX3](http://blast.ncbi.nlm.nih.gov/Blast.cgi#alnHdr_60476416) |
| LjSGA_022785.1 | 2.58 | 5.00E-05 | TALE | homeobox protein Meis1 | [K15613](http://www.genome.jp/dbget-bin/www_bget?ko:K15613) | [homeobox protein knotted-1-like 1](http://blast.ncbi.nlm.nih.gov/Blast.cgi#alnHdr_702443712) |
| chr1.CM0105.740.r2.a | 3.38 | 5.00E-05 | WRKY | probable WRKY TF 52 | [K16225](http://www.genome.jp/dbget-bin/www_bget?ko:K16225) | [probable WRKY TF 53-like](http://blast.ncbi.nlm.nih.gov/Blast.cgi#alnHdr_356573221) |
| chr4.CM1622.200.r2.a | 2.76 | 5.00E-05 | WRKY | WRKY TF 33 | [K13424](http://www.genome.jp/dbget-bin/www_bget?ko:K13424) | [Putative WRKY TF 13](http://blast.ncbi.nlm.nih.gov/Blast.cgi#alnHdr_734395770) |
| LjSGA_012799.2 | 2.10 | 5.00E-05 | WRKY | WRKY TF 33 | [K13424](http://www.genome.jp/dbget-bin/www_bget?ko:K13424) | probable WRKY TF 50 |
| chr5.CM0311.260.r2.m | 2.33 | 5.00E-05 | bHLH | NA | NA | TF bHLH111-like isoform X2 |
| chr5.CM0180.280.r2.m | 2.30 | 5.00E-05 | C2H2 | NA | NA | zinc finger protein ZAT11-like |
| LjSGA_020980.2 | 3.94 | 5.00E-05 | C2H2 | NA | NA | zinc finger protein ZAT11-like |
| gene=XLOC_019119 | 3.53 | 1.50E-03 | MYB | NA | NA | TF MYB86 |
| chr3.CM0590.350.r2.d | 3.23 | 5.00E-05 | NAC | NA | NA | NAC domain protein |
| chr4.LjT06B21.210.r2.d | 3.15 | 5.00E-05 | NAC | NA | NA | NAC-domain protein |
| LjSGA_027787.1 | 3.48 | 5.00E-05 | NAC | NA | NA | NAC domain-containing protein 43 |
| LjSGA_036303.1 | 3.36 | 5.00E-05 | NAC | NA | NA | NAC domain protein NAC3 |
| LjSGA_068911.1 | 2.55 | 1.24E-02 | NAC | NA | NA | protein CUP-SHAPED COTYLEDON 3-like |
| gene=XLOC_012455 | 2.00 | 5.00E-05 | WRKY | NA | NA | Putative WRKY TF 46 |
| chr2.CM1835.100.r2.m | 3.01 | 5.00E-05 | ZF-HD | NA | NA | Mini zinc finger 2 isoform 1 |
| chr4.CM0087.50.r2.m | 2.01 | 5.00E-05 | ZF-HD | NA | NA | ZF-HD homeobox protein At4g24660-like |
| LjSGA_066816.1 | 2.42 | 2.80E-03 | ZF-HD | NA | NA | ZF-HD homeobox protein At4g24660-like |
