# Supplementary material for: Transcriptome Response Mediated by Cold Stress in Lotus japonicus
Source: Front Plant Sci. 2016 Mar 30;7:374. doi: 10.3389/fpls.2016.00374 (PMC4811897; doi:10.3389/fpls.2016.00374)
Supplement: Supplementary file 4 [file Table4.DOCX]

Supplementary Material

**Transcriptome response mediated by cold stress in *Lotus japonicus.***

Pablo Ignacio Calzadilla, Santiago Javier Maiale, Oscar Adolfo^*^ Ruiz and Francisco José Escaray.

*** Correspondence:** ruiz@intech.gov.ar

**Supplementary Table 4. Up-regulated genes with no annotation in the *L. japonicus* genome.** A BLASTx search was done, and the best hits with an E-value<0.001 are shown.

| **Transcript** | **BLASTx Hit** | **E-value** |
| --- | --- | --- |
| gene=XLOC_001513 | >ref\|XP_004506381.1\| PREDICTED: enzymatic polyprotein-like [Cicer arietinum] | 3.00E-113 |
| gene=XLOC_015856 | >ref\|XP_010026916.1\| PREDICTED: salicylate carboxymethyltransferase-like [Eucalyptus grandis] | 7.00E-05 |
| gene=XLOC_023605 | >ref\|XP_008226782.1\| PREDICTED: putative ribonuclease H protein At1g65750 [Prunus mume] | 6.00E-22 |
| gene=XLOC_007027 | >ref\|XP_007039706.1\| Non-LTR retroelement reverse transcriptase [Theobroma cacao] | 3.00E-16 |
| gene=XLOC_011264 | >ref\|XP_006599883.1\| PREDICTED: putative ribonuclease H protein At1g65750-like [Glycine max] | 6.00E-80 |
| gene=XLOC_024127 | >ref\|XP_006594475.1\| PREDICTED: cytokinesis protein sepA-like isoform X1 [Glycine max] | 2.00E-09 |
| gene=XLOC_011184 | >ref\|XP_004301685.1\| PREDICTED: putative ribonuclease H protein At1g65750-like [Fragaria vesca subsp. vesca] | 3.00E-09 |
| gene=XLOC_022156 | >ref\|XP_004501043.1\| PREDICTED: elongation of fatty acids protein A-like [Cicer arietinum] | 3.00E-151 |
| gene=XLOC_022911 | >ref\|XP_003546171.1\| PREDICTED: ubiquinol oxidase 4 chloroplastic/chromoplastic isoform X1 [Glycine max] | 2.00E-67 |
| gene=XLOC_039851 | >ref\|XP_003540091.1\| PREDICTED: stem-specific protein TSJT1-like [Glycine max] | 7.00E-07 |
| gene=XLOC_015126 | >ref\|XP_003522822.1\| PREDICTED: SUN domain-containing ossification factor-like [Glycine max] | 3.00E-40 |
| gene=XLOC_011291 | >ref\|NP_001235345.1\| receptor-like protein kinase precusor-like protein [Glycine max] | 3.00E-05 |
| gene=XLOC_007159 | >gb\|KHN45935.1\| Prostaglandin E synthase 2 [Glycine soja] | 2.00E-21 |
| gene=XLOC_007861 | >gb\|KHN34192.1\| Putative mitochondrial chaperone BCS1-B [Glycine soja] | 0 |
| gene=XLOC_020432 | >gb\|KHN29805.1\| Putative serine/threonine-protein kinase [Glycine soja] | 4.00E-55 |
| gene=XLOC_020965 | >gb\|KHN24710.1\| Bromodomain-containing protein 9 partial [Glycine soja] | 6.00E-06 |
| gene=XLOC_014100 | >gb\|KHN20554.1\| Putative sucrose-phosphate synthase 4 [Glycine soja] | 1.00E-21 |
| gene=XLOC_013451 | >gb\|KHN18980.1\| Ubiquitin carboxyl-terminal hydrolase 13 [Glycine soja] | 9.00E-13 |
| gene=XLOC_019119 | >gb\|KHN16792.1\| TF MYB86 [Glycine soja] | 1.00E-30 |
| gene=XLOC_012455 | >gb\|KHN15951.1\| Putative WRKY TF 46 [Glycine soja] | 1.00E-18 |
| gene=XLOC_015011 | >gb\|KHN13665.1\| Retrovirus-related Pol polyprotein from transposon TNT 1-94 partial [Glycine soja] | 2.00E-41 |
| gene=XLOC_019761 | >gb\|KHN08401.1\| Protein FAR1-RELATED SEQUENCE 12 [Glycine soja] | 3.00E-05 |
| gene=XLOC_021228 | >gb\|KHN04950.1\| Pentatricopeptide repeat-containing protein [Glycine soja] | 4.00E-67 |
| gene=XLOC_015174 | >gb\|KHN02162.1\| Protein FAR1-RELATED SEQUENCE 6 partial [Glycine soja] | 8.00E-47 |
| gene=XLOC_007310 | >gb\|KEH41540.1\| transmembrane protein putative [Medicago truncatula] | 3.00E-14 |
| gene=XLOC_015171 | >gb\|KEH40110.1\| DUF594 family protein [Medicago truncatula] | 7.00E-154 |
| gene=XLOC_023899 | >gb\|AES65133.2\| cellulose synthase-like protein [Medicago truncatula] | 0 |
| gene=XLOC_011268 | >gb\|ACL97387.1\| Gag-Pol polyprotein [Lotus japonicus] | 0 |
| gene=XLOC_018989 | >gb\|ABE80156.1\| Ribonuclease H [Medicago truncatula] | 3.00E-50 |
| gene=XLOC_007309 | >gb\|ABA91380.1\| retrotransposon protein putative Ty1-copia subclass [Oryza | 8.00E-76 |
| gene=XLOC_015010 | >gb\|AAX92941.1\| retrotransposon protein putative Ty1-copia sub-class [Oryza sativa Japonica Group] | 7.00E-91 |
| gene=XLOC_018954 | >gb\|AAX92941.1\| retrotransposon protein putative Ty1-copia sub-class [Oryza sativa Japonica Group] | 0 |
| gene=XLOC_011125 | >gb\|AAG60117.1\|AC073555_1 copia-type polyprotein putative [Arabidopsis thaliana] | 3.00E-46 |
| gene=XLOC_016989 | >gb\|AAA97907.1\| cysteine proteinase inhibitor [Glycine max] | 2.00E-13 |
