# Supplementary material for: Transcriptome Response Mediated by Cold Stress in Lotus japonicus
Source: Front Plant Sci. 2016 Mar 30;7:374. doi: 10.3389/fpls.2016.00374 (PMC4811897; doi:10.3389/fpls.2016.00374)
Supplement: Supplementary file 5 [file Table5.DOCX]

Supplementary Material

**Transcriptome response mediated by cold stress in *Lotus japonicus.***

Pablo Ignacio Calzadilla, Santiago Javier Maiale, Oscar Adolfo^*^ Ruiz and Francisco José Escaray.

*** Correspondence:** ruiz@intech.gov.ar

**Supplementary Table 5. Down-regulated genes with no annotation in the *L. japonicus* genome.** A BLASTx search was done, and the best hits with an E-value<0.001 are shown.

| **Transcript** | **BLASTx Hit** | **E-value** |
| --- | --- | --- |
| gene=XLOC_007162 | >emb\|CAC44140.1\| putative polyprotein [Cicer arietinum] | 3.00E-20 |
| gene=XLOC_015180 | >emb\|CAB65284.1\| putative wound-induced protein [Medicago sativa subsp. x varia] | 1.00E-24 |
| gene=XLOC_011186 | >emb\|CAA73364.1\| Pge1 protein [Lotus japonicus] | 2.00E-14 |
| gene=XLOC_007062 | >gb\|AER13160.1\| putative non-LTR retroelement [Phaseolus vulgaris] | 1.00E-20 |
| gene=XLOC_007243 | >gb\|KHN19400.1\| Protein FAR1-RELATED SEQUENCE 5. partial [Glycine soja] | 4.00E-89 |
| gene=XLOC_025814 | >gb\|KHN15506.1\| Putative ribonuclease H protein. partial [Glycine soja] | 1.00E-26 |
| gene=XLOC_005048 | >gb\|KHN08389.1\| Nuclear pore complex protein Nup205 [Glycine soja] | 4.00E-05 |
| gene=XLOC_008347 | >gb\|KHN41902.1\| hypothetical protein glysoja_003642 [Glycine soja] | 5.00E-25 |
| gene=XLOC_015080 | >gb\|KHN33983.1\| Pectinesterase inhibitor 1. partial [Glycine soja] | 3.00E-22 |
| gene=XLOC_001813 | >gb\|KHN27546.1\| LINE-1 reverse transcriptase like. partial [Glycine soja] | 3.00E-100 |
| gene=XLOC_018911 | >ref\|XP_004505098.1\| PREDICTED: receptor-like protein kinase HSL1-like [Cicer arietinum] | 3.00E-13 |
| gene=XLOC_024442 | >ref\|XP_003614385.1\| RRNA intron-encoded homing endonuclease [Medicago truncatula] | 1.00E-42 |
| gene=XLOC_014959 | >ref\|XP_003598700.1\| Serine/threonine protein kinase [Medicago truncatula] | 7.00E-07 |
| gene=XLOC_015142 | >ref\|XP_003550174.1\| PREDICTED: monothiol glutaredoxin-S2-like [Glycine max] | 3.00E-49 |
| gene=XLOC_011074 | >ref\|XP_003543162.1\| PREDICTED: carbon catabolite repressor protein 4 homolog 5-like isoform X1 [Glycine max] | 2.00E-04 |
| gene=XLOC_003911 | >ref\|XP_003529624.1\| PREDICTED: mitochondrial arginine transporter BAC2-like [Glycine max] | 3.00E-04 |
| gene=XLOC_004510 | >ref\|XP_006577118.1\| PREDICTED: cohesin subunit SA-1 isoform X2 [Glycine max] | 4.00E-12 |
| gene=XLOC_021243 | >ref\|XP_008450744.1\| PREDICTED: sec-independent protein translocase protein TATA. | 2.00E-31 |
| gene=XLOC_001433 | >ref\|XP_006574043.1\| PREDICTED: putative ribonuclease H protein At1g65750-like [Glycine max] | 9.00E-20 |
| gene=XLOC_019818 | >ref\|XP_010647371.1\| PREDICTED: protein FAR1-RELATED SEQUENCE 5-like [Vitis vinifera] | 8.00E-12 |
| gene=XLOC_019819 | >ref\|XP_010644068.1\| PREDICTED: protein FAR1-RELATED SEQUENCE 5-like [Vitis vinifera] | 6.00E-129 |
