# Supplementary material for: Transcriptome Response Mediated by Cold Stress in Lotus japonicus
Source: Front Plant Sci. 2016 Mar 30;7:374. doi: 10.3389/fpls.2016.00374 (PMC4811897; doi:10.3389/fpls.2016.00374)
Supplement: Supplementary file 6 [file Image1.PDF]

# *Supplementary Material*

# Transcriptome response mediated by cold stress in *Lotus japonicus*

Pablo Ignacio Calzadilla, Santiago Javier Maiale, Oscar Adolfo* Ruiz and Francisco José Escaray.

*** Correspondence:** ruiz@intech.gov.ar

## 1 Supplementary Figures and Tables

### 1.1 Supplementary Figures

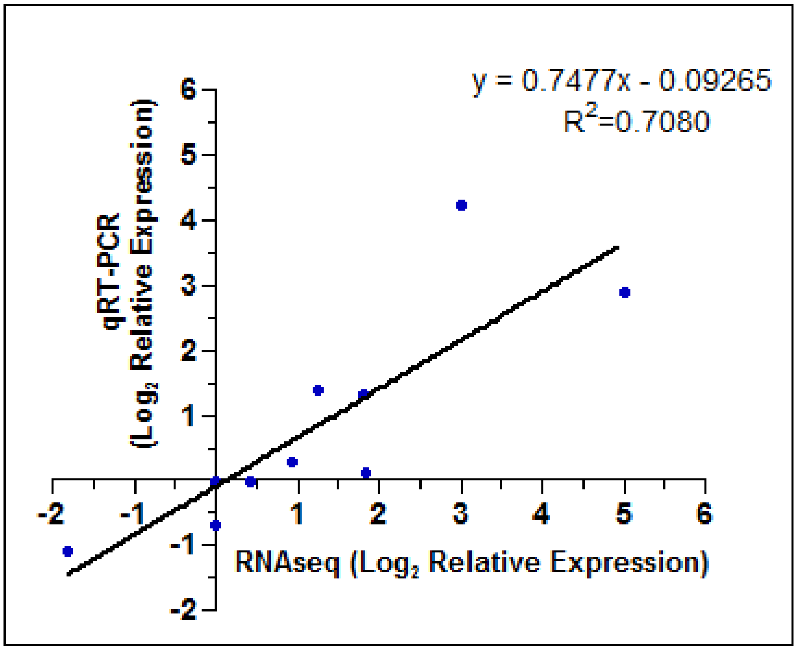


**Supplementary Figure 1.** Comparison of RNAseq and quantitative real-time PCR data for 10 selected genes. Symbols represent $Log_2$ transformation of mean expression levels relative to control treatments.

## 1.2 Supplementary Tables

**Supplementary Table 1. Primers used for qRT-PCR.**

| Gene ID | Forward primer | Reverse primer |
|---|---|---|
| *chr1.CM0104.550.r2.m* | GTTGGACAACCTCGGATTGG | CTGGCCTTCTGTGCATTGCT |
| *chr3.CM0091.1980.r2.m* | GCATTCTGGCCATTTTGGA | GCCCACTTGCTCAAGCTTCT |
| *chr1.CM0113.680.r2.d* | CACCGATCTGACAGCAGCTCTA | CATCTGTACTCTTCACCTCTTTCTTTTC |
| *chr5.CM0180.280.r2.m* | CAGCAGGGAGGTCCATATGAA | GGAACTTCCGGTTGCATGTC |
| *chr5.CM0148.540.r2.m* | GATGTTTCTGACCCCTCAGGAT | CCTCTGCTACCTTCCCATAAACTG |
| *chr4.CM0126.2020.r2.a* | CGGAAGCAGGTTCCTCTAGCT | CGGTACACAGGGTGCCTTGT |
| *chr4.CM0126.2110.r2.a* | GAAGTCCGCTTGCCTCAACT | TTCCGTTGCCACTCTCCTAAG |
| *chr5.CM0359.290.r2.m* | TGAAACGCGACACCCTACCT | GAGCCACACACGCGCTTT |
| *LjSGA_021886.2* | TGAGCTTGTGAAGGTTGG | AACAGGGAGTTGACAAATCT |
| *chr1.CM0378.230.r2.m* | AAGATGGAGAGGGATATGG | GTCTTGTTCTCACGCTTT |
| *LjSGA_063085.1* | ATACAACTACAGCGTCAT | GCAATCAATTTGGACTCA |

**Supplementary Table 2. Differentially expressed genes classified under the organismal system category of the KEGG Pathway Database.**

| Kegg Pathways DB | | Gene ID | $Log_2$ FC | p value | Functional annotation | Kegg ID |
|---|---|---|---|---|---|---|
| **Organismal system** | **Endocryne system** | *chr1.CM0637.320.r2.a* | 2.31 | 1.50E-04 | heat shock 70kDa protein 1/8 | K03283 |
| | | *chr3.CM0786.370.r2.a* | 3.74 | 5.00E-05 | heat shock 70kDa protein 1/8 | K03283 |
| | | *chr4.CM0179.510.r2.d* | 3.94 | 5.00E-05 | heat shock 70kDa protein 1/8 | K03283 |
| | | *LjSGA_034228.1* | 3.83 | 5.00E-05 | heat shock 70kDa protein 1/8 | K03283 |
| | | *LjSGA_076792.1* | 4.05 | 5.00E-05 | heat shock 70kDa protein 1/8 | K03283 |
| | | *LjSGA_015689.1* | -2.68 | 5.00E-05 | heat shock 70kDa protein 1/8 | K03283 |
| | **Environmental adaptation** | *chr1.CM0121.30.r2.m* | 2.91 | 5.00E-05 | calmodulin | K02183 |
| | | *LjT47J13.70.r2.a* | 2.68 | 5.00E-05 | zinc finger protein CONSTANS | K12135 |
| | | *chr1.CM1868.80.r2.a* | 4.16 | 5.00E-05 | Dof zinc finger protein DOF5.5 | K16222 |
| | | *chr1.CM0122.1220.r2.m* | 2.34 | 5.00E-05 | phytochrome-interacting factor 3 | K12126 |
| | | *chr3.CM0155.170.r2.d* | 3.17 | 5.00E-05 | zinc finger protein CONSTANS | K12135 |
| | | *chr4.CM0087.500.r2.m* | 3.18 | 5.00E-05 | pseudo-response regulator 1 | K12127 |
| | | *chr4.CM0042.1360.r2.m* | 5.08 | 5.00E-05 | flavin-binding kelch repeat F-box protein 1 | K12116 |
| | | *chr5.CM0456.540.r2.m* | 4.10 | 5.00E-05 | MYB-related TF LHY | K12133 |
| | | *LjSGA_092681.1* | 3.99 | 5.00E-05 | pseudo-response regulator 5 | K12130 |
| | | *chr4.CM0042.2080.r2.m* | -4.03 | 5.00E-05 | protein FLOWERING LOCUS T | K16223 |

| | | | | |
|---|---|---|---|---|
| *LjSGA_100666.1* | -3.14 | 5.00E-05 | zinc finger protein CONSTANS | K12135 |
| *chr3.CM0106.190.r2.m* | 2.45 | 5.00E-05 | calcium-binding protein CML | K13448 |
| *LjT06N06.390.r2.d* | 2.78 | 5.00E-05 | brassinosteroid insensitive 1-associated receptor kinase 1 [EC:2.7.10.1 2.7.11.1] | K13416 |
| *chr1.CM0104.1860.r2.d* | 2.30 | 5.00E-05 | disease resistance protein RPS4 | K16226 |
| *chr1.CM0393.520.r2.d* | 3.07 | 5.00E-05 | disease resistance protein RPS4 | K16226 |
| *chr1.CM0105.740.r2.a* | 3.38 | 5.00E-05 | probable WRKY TF 52 | K16225 |
| *chr2.LjB15M17.60.r2.m* | 3.04 | 5.00E-05 | LRR receptor-like serine/threonine-protein kinase FLS2 [EC:2.7.11.1] | K13420 |
| *chr2.CM0020.160.r2.d* | 2.26 | 5.00E-05 | disease resistance protein RPM1 | K13457 |
| *chr4.CM0161.190.r2.d* | 2.81 | 5.50E-04 | brassinosteroid insensitive 1-associated receptor kinase 1 [EC:2.7.10.1 2.7.11.1] | K13416 |
| *chr4.CM1622.200.r2.a* | 2.76 | 5.00E-05 | WRKY TF 33 | K13424 |
| *chr5.CM0148.470.r2.m* | 2.26 | 5.00E-05 | LRR receptor-like serine/threonine-protein kinase FLS2 [EC:2.7.11.1] | K13420 |
| *chr5.CM0456.520.r2.m* | 2.31 | 5.00E-05 | LRR receptor-like serine/threonine-protein kinase FLS2 [EC:2.7.11.1] | K13420 |
| *chr6.CM0679.520.r2.d* | 2.63 | 5.00E-05 | disease resistance protein RPS4 | K16226 |
| *LjSGA_012799.2* | 2.10 | 5.00E-05 | WRKY TF 33 | K13424 |
| *LjSGA_017567.1* | 2.67 | 5.00E-05 | LRR receptor-like serine/threonine-protein kinase EFR [EC:2.7.11.1] | K13428 |
| *LjSGA_023624.0.1* | 3.81 | 8.65E-03 | calcium-binding protein CML | K13448 |
| *LjSGA_033122.2* | 6.39 | 5.00E-05 | molecular chaperone HtpG | K04079 |
| *LjSGA_033246.1* | 6.00 | 5.00E-05 | molecular chaperone HtpG | K04079 |

| | | | | | |
|---|---|---|---|---|---|
| | *LjSGA_034276.1* | 2.51 | 5.00E-05 | respiratory burst oxidase [EC:1.6.3.- 1.11.1.-] | K13447 |
| | *LjSGA_063791.1* | 2.03 | 6.85E-03 | calcium-binding protein CML | K13448 |
| | *LjSGA_064721.0.1* | 2.00 | 5.00E-05 | molecular chaperone HtpG | K04079 |
| | *LjSGA_074430.1* | 2.56 | 5.00E-05 | calcium-binding protein CML | K13448 |
| | *LjSGA_113688.1* | 2.29 | 2.70E-03 | LRR receptor-like serine/threonine-protein kinase EFR [EC:2.7.11.1] | K13428 |
| | *chr3.CM0208.290.r2.m* | -2.15 | 5.00E-05 | TF MYC2 | K13422 |
| | *LjSGA_061207.1* | -2.10 | 5.00E-05 | disease resistance protein RPS4 | K16226 |
| | *LjSGA_134572.1* | -2.47 | 5.00E-05 | pathogenesis-related protein 1 | K13449 |
| **Immune system** | *chr1.CM0163.10.r2.d* | 2.15 | 1.65E-03 | interleukin-1 receptor-associated kinase 4 [EC:2.7.11.1] | K04733 |
| | *chr2.CM0008.460.r2.m* | 2.57 | 5.00E-05 | interleukin-1 receptor-associated kinase 4 [EC:2.7.11.1] | K04733 |
| | *chr3.CM0005.410.r2.m* | 2.01 | 5.00E-05 | interleukin-1 receptor-associated kinase 4 [EC:2.7.11.1] | K04733 |
| | *chr4.CM0161.220.r2.d* | 2.22 | 5.00E-05 | interleukin-1 receptor-associated kinase 4 [EC:2.7.11.1] | K04733 |
| | *chr4.CM0042.860.r2.m* | 2.97 | 5.00E-05 | interleukin-1 receptor-associated kinase 4 [EC:2.7.11.1] | K04733 |
| | *chr5.CM0328.1020.r2.d* | 2.54 | 5.00E-05 | interleukin-1 receptor-associated kinase 4 [EC:2.7.11.1] | K04733 |
| | *LjSGA_038530.1* | 4.10 | 6.40E-03 | interleukin-1 receptor-associated kinase 4 [EC:2.7.11.1] | K04733 |
| | *LjSGA_086627.1* | 2.04 | 5.00E-05 | interleukin-1 receptor-associated kinase 4 [EC:2.7.11.1] | K04733 |
| | *chr3.CM0005.420.r2.m* | -2.22 | 5.00E-05 | interleukin-1 receptor-associated kinase 4 [EC:2.7.11.1] | K04733 |

**Supplementary Table 3. Cold stress up-regulated TFs.** Sequences labeled as NA were not annotated in the KEGG Database. Putative novel TFs are underlined.

| Gene Accession | Log2 FC | p-value | TF Family | KEGG Annotation | KO | Blastx Results |
|---|---|---|---|---|---|---|
| chr2.CM0060.290.r2.a | 2.69 | 5.00E-05 | AP2/ERF | EREBP-like factor | K09286 | Ethylene-responsive TF ERF061 |
| chr4.CM0536.270.r2.d | 2.39 | 5.00E-05 | AP2/ERF | EREBP-like factor | K09286 | ethylene-responsive TF ERF118-like |
| chr5.CM0494.320.r2.m | 2.33 | 2.80E-03 | AP2/ERF | ethylene-responsive TF 1 | K14516 | ethylene-responsive TF 1B-like |
| chr5.CM0052.670.r2.d | 2.07 | 5.00E-05 | AP2/ERF | EREBP-like factor | K09286 | ethylene-responsive TF ERF110-like |
| LjSGA_025266.1 | 3.33 | 5.00E-05 | AP2/ERF | EREBP-like factor | K09286 | ethylene-responsive TF ERF053-like |
| LjSGA_034198.1 | 2.63 | 5.00E-05 | AP2/ERF | EREBP-like factor | K09286 | ethylene-responsive TF ERF053-like |
| chr4.CM0087.500.r2.m | 3.18 | 5.00E-05 | ARR-B/CO-Like | pseudo-response regulator 1 | K12127 | Two-component response regulator-like APRR1 |
| LjSGA_092681.1 | 3.99 | 5.00E-05 | ARR-B | pseudo-response regulator 5 | K12130 | Two-component response regulator-like PRR95 |
| chr1.CM0122.1220.r2.m | 2.34 | 5.00E-05 | bHLH | phytochrome-interacting factor 3 | K12126 | TF PIF3-like |
| chr4.CM0307.390.r2.d | 3.20 | 5.00E-05 | CAMTA | myosin V | K10357 | Calmodulin-binding transcription activator 2 |
| chr2.CM0021.1970.r2.m | 2.12 | 5.00E-05 | C2H2 | KRAB domain-containing zinc finger protein | K09228 | Zinc finger protein ZAT9 |

| | | | | | | |
|---|---|---|---|---|---|---|
| LjT47J13.70.r2.a | 2.68 | 5.00E-05 | DBB | zinc finger protein CONSTANS | K12135 | B-box type zinc finger protein |
| chr3.CM0155.170.r2.d | 3.17 | 5.00E-05 | DBB | zinc finger protein CONSTANS | K12135 | Zinc finger protein CONSTANS-LIKE 14 |
| chr1.CM1868.80.r2.a | 4.16 | 5.00E-05 | Dof | Dof zinc finger protein DOF5.5 | K16222 | dof zinc finger protein DOF5.4 |
| LjT15C06.80.r2.m | 2.19 | 5.00E-05 | FAR1 | zinc finger SWIM domain-containing protein 3 | K17604 | protein FAR1-RELATED SEQUENCE 5-like |
| chr1.CM0133.500.r2.m | 3.55 | 5.00E-05 | HSF | heat shock TF. other eukaryote | K09419 | heat shock factor protein HSF30-like |
| chr1.CM0122.1190.r2.m | 2.07 | 5.00E-05 | MYB | myb proto-oncogene protein. Plant | K09422 | TF MYB86 |
| chr2.CM0435.1360.r2.m | 2.57 | 2.50E-04 | MYB | myb proto-oncogene protein. Plant | K09422 | TF MYB44 |
| chr4.CM0042.700.r2.m | 2.45 | 5.00E-05 | MYB | myb proto-oncogene protein. Plant | K09422 | TF MYB21 |
| chr2.CM0168.360.r2.d | 2.27 | 3.60E-03 | MYB_related | myb proto-oncogene protein. Plant | K09422 | telomere repeat-binding factor 2-like |
| chr4.CM0126.410.r2.d | 2.44 | 4.60E-03 | MYB_related | myb proto-oncogene protein. Plant | K09422 | protein ODORANT1 |
| chr5.CM0456.540.r2.m | 4.10 | 5.00E-05 | MYB_related | MYB-related TF LHY | K12133 | TF ASG4-like isoform X1 |
| chr6.CM0367.750.r2.m | 2.27 | 5.00E-05 | TALE | homeobox protein Meis1 | K15613 | Knox class 1 protein |
| LjSGA_010170.1 | 2.10 | 7.80E-03 | TALE | homeobox protein homothorax | K16672 | homeobox knotted-1-like protein KNOX3 |
| LjSGA_022785.1 | 2.58 | 5.00E-05 | TALE | homeobox protein Meis1 | K15613 | homeobox protein knotted-1-like 1 |
| chr1.CM0105.740.r2.a | 3.38 | 5.00E-05 | WRKY | probable WRKY TF 52 | K16225 | probable WRKY TF 53-like |

| | | | | | | |
|---|---|---|---|---|---|---|
| chr4.CM1622.200.r2.a | 2.76 | 5.00E-05 | WRKY | WRKY TF 33 | K13424 | Putative WRKY TF 13 |
| LjSGA_012799.2 | 2.10 | 5.00E-05 | WRKY | WRKY TF 33 | K13424 | probable WRKY TF 50 |
| chr5.CM0311.260.r2.m | 2.33 | 5.00E-05 | bHLH | NA | NA | TF bHLH111-like isoform X2 |
| chr5.CM0180.280.r2.m | 2.30 | 5.00E-05 | C2H2 | NA | NA | zinc finger protein ZAT11-like |
| LjSGA_020980.2 | 3.94 | 5.00E-05 | C2H2 | NA | NA | zinc finger protein ZAT11-like |
| gene=XLOC_019119 | 3.53 | 1.50E-03 | MYB | NA | NA | TF MYB86 |
| chr3.CM0590.350.r2.d | 3.23 | 5.00E-05 | NAC | NA | NA | NAC domain protein |
| chr4.LjT06B21.210.r2.d | 3.15 | 5.00E-05 | NAC | NA | NA | NAC-domain protein |
| LjSGA_027787.1 | 3.48 | 5.00E-05 | NAC | NA | NA | NAC domain-containing protein 43 |
| LjSGA_036303.1 | 3.36 | 5.00E-05 | NAC | NA | NA | NAC domain protein NAC3 |
| LjSGA_068911.1 | 2.55 | 1.24E-02 | NAC | NA | NA | protein CUP-SHAPED COTYLEDON 3-like |
| gene=XLOC_012455 | 2.00 | 5.00E-05 | WRKY | NA | NA | Putative WRKY TF 46 |
| chr2.CM1835.100.r2.m | 3.01 | 5.00E-05 | ZF-HD | NA | NA | Mini zinc finger 2 isoform 1 |
| chr4.CM0087.50.r2.m | 2.01 | 5.00E-05 | ZF-HD | NA | NA | ZF-HD homeobox protein At4g24660-like |
| LjSGA_066816.1 | 2.42 | 2.80E-03 | ZF-HD | NA | NA | ZF-HD homeobox protein At4g24660-like |

**Supplementary Table 4. Up-regulated genes with no annotation in the *L. japonicus* genome.** A BLASTx search was done, and the best hits with an E-value<0.001 are shown.

| Transcript | BLASTx Hit | E-value |
|---|---|---|
| gene=XLOC_001513 | >ref\|XP_004506381.1\| PREDICTED: enzymatic polyprotein-like [Cicer arietinum] | 3.00E-113 |
| gene=XLOC_015856 | >ref\|XP_010026916.1\| PREDICTED: salicylate carboxymethyltransferase-like [Eucalyptus grandis] | 7.00E-05 |
| gene=XLOC_023605 | >ref\|XP_008226782.1\| PREDICTED: putative ribonuclease H protein At1g65750 [Prunus mume] | 6.00E-22 |
| gene=XLOC_007027 | >ref\|XP_007039706.1\| Non-LTR retroelement reverse transcriptase [Theobroma cacao] | 3.00E-16 |
| gene=XLOC_011264 | >ref\|XP_006599883.1\| PREDICTED: putative ribonuclease H protein At1g65750-like [Glycine max] | 6.00E-80 |
| gene=XLOC_024127 | >ref\|XP_006594475.1\| PREDICTED: cytokinesis protein sepA-like isoform X1 [Glycine max] | 2.00E-09 |
| gene=XLOC_011184 | >ref\|XP_004301685.1\| PREDICTED: putative ribonuclease H protein At1g65750-like [Fragaria vesca subsp. vesca] | 3.00E-09 |
| gene=XLOC_022156 | >ref\|XP_004501043.1\| PREDICTED: elongation of fatty acids protein A-like [Cicer arietinum] | 3.00E-151 |
| gene=XLOC_022911 | >ref\|XP_003546171.1\| PREDICTED: ubiquinol oxidase 4 chloroplastic/chromoplastic isoform X1 [Glycine max] | 2.00E-67 |
| gene=XLOC_039851 | >ref\|XP_003540091.1\| PREDICTED: stem-specific protein TSJT1-like [Glycine max] | 7.00E-07 |
| gene=XLOC_015126 | >ref\|XP_003522822.1\| PREDICTED: SUN domain-containing ossification factor-like [Glycine max] | 3.00E-40 |
| gene=XLOC_011291 | >ref\|NP_001235345.1\| receptor-like protein kinase precusor-like protein [Glycine max] | 3.00E-05 |
| gene=XLOC_007159 | >gb\|KHN45935.1\| Prostaglandin E synthase 2 [Glycine soja] | 2.00E-21 |
| gene=XLOC_007861 | >gb\|KHN34192.1\| Putative mitochondrial chaperone BCS1-B [Glycine soja] | 0 |
| gene=XLOC_020432 | >gb\|KHN29805.1\| Putative serine/threonine-protein kinase [Glycine soja] | 4.00E-55 |

| | | |
|---|---|---|
| gene=XLOC_020965 | >gb\|KHN24710.1\| Bromodomain-containing protein 9  partial [Glycine soja] | 6.00E-06 |
| gene=XLOC_014100 | >gb\|KHN20554.1\| Putative sucrose-phosphate synthase 4 [Glycine soja] | 1.00E-21 |
| gene=XLOC_013451 | >gb\|KHN18980.1\| Ubiquitin carboxyl-terminal hydrolase 13 [Glycine soja] | 9.00E-13 |
| gene=XLOC_019119 | >gb\|KHN16792.1\| TF MYB86 [Glycine soja] | 1.00E-30 |
| gene=XLOC_012455 | >gb\|KHN15951.1\| Putative WRKY TF 46 [Glycine soja] | 1.00E-18 |
| gene=XLOC_015011 | >gb\|KHN13665.1\| Retrovirus-related Pol polyprotein from transposon TNT 1-94  partial [Glycine soja] | 2.00E-41 |
| gene=XLOC_019761 | >gb\|KHN08401.1\| Protein FAR1-RELATED SEQUENCE 12 [Glycine soja] | 3.00E-05 |
| gene=XLOC_021228 | >gb\|KHN04950.1\| Pentatricopeptide repeat-containing protein [Glycine soja] | 4.00E-67 |
| gene=XLOC_015174 | >gb\|KHN02162.1\| Protein FAR1-RELATED SEQUENCE 6  partial [Glycine soja] | 8.00E-47 |
| gene=XLOC_007310 | >gb\|KEH41540.1\| transmembrane protein  putative [Medicago truncatula] | 3.00E-14 |
| gene=XLOC_015171 | >gb\|KEH40110.1\| DUF594 family protein [Medicago truncatula] | 7.00E-154 |
| gene=XLOC_023899 | >gb\|AES65133.2\| cellulose synthase-like protein [Medicago truncatula] | 0 |
| gene=XLOC_011268 | >gb\|ACL97387.1\| Gag-Pol polyprotein [Lotus japonicus] | 0 |
| gene=XLOC_018989 | >gb\|ABE80156.1\| Ribonuclease H [Medicago truncatula] | 3.00E-50 |
| gene=XLOC_007309 | >gb\|ABA91380.1\| retrotransposon protein  putative  Ty1-copia subclass [Oryza | 8.00E-76 |
| gene=XLOC_015010 | >gb\|AAX92941.1\| retrotransposon protein putative  Ty1-copia sub-class [Oryza sativa Japonica Group] | 7.00E-91 |
| gene=XLOC_018954 | >gb\|AAX92941.1\| retrotransposon protein putative  Ty1-copia sub-class [Oryza sativa Japonica Group] | 0 |
| gene=XLOC_011125 | >gb\|AAG60117.1\|AC073555_1 copia-type polyprotein  putative [Arabidopsis thaliana] | 3.00E-46 |
| gene=XLOC_016989 | >gb\|AAA97907.1\| cysteine proteinase inhibitor [Glycine max] | 2.00E-13 |

**Supplementary Table 5. Down-regulated genes with no annotation in the *L. japonicus* genome.** A BLASTx search was done, and the best hits with an E-value<0.001 are shown.

| Transcript | BLASTx Hit | E-value |
|---|---|---|
| gene=XLOC_007162 | >emb\|CAC44140.1\| putative polyprotein [Cicer arietinum] | 3.00E-20 |
| gene=XLOC_015180 | >emb\|CAB65284.1\| putative wound-induced protein [Medicago sativa subsp. x varia] | 1.00E-24 |
| gene=XLOC_011186 | >emb\|CAA73364.1\| Pge1 protein [Lotus japonicus] | 2.00E-14 |
| gene=XLOC_007062 | >gb\|AER13160.1\| putative non-LTR retroelement [Phaseolus vulgaris] | 1.00E-20 |
| gene=XLOC_007243 | >gb\|KHN19400.1\| Protein FAR1-RELATED SEQUENCE 5. partial [Glycine soja] | 4.00E-89 |
| gene=XLOC_025814 | >gb\|KHN15506.1\| Putative ribonuclease H protein. partial [Glycine soja] | 1.00E-26 |
| gene=XLOC_005048 | >gb\|KHN08389.1\| Nuclear pore complex protein Nup205 [Glycine soja] | 4.00E-05 |
| gene=XLOC_008347 | >gb\|KHN41902.1\| hypothetical protein glysoja_003642 [Glycine soja] | 5.00E-25 |
| gene=XLOC_015080 | >gb\|KHN33983.1\| Pectinesterase inhibitor 1. partial [Glycine soja] | 3.00E-22 |
| gene=XLOC_001813 | >gb\|KHN27546.1\| LINE-1 reverse transcriptase like. partial [Glycine soja] | 3.00E-100 |
| gene=XLOC_018911 | >ref\|XP_004505098.1\| PREDICTED: receptor-like protein kinase HSL1-like [Cicer arietinum] | 3.00E-13 |
| gene=XLOC_024442 | >ref\|XP_003614385.1\| RRNA intron-encoded homing endonuclease [Medicago truncatula] | 1.00E-42 |
| gene=XLOC_014959 | >ref\|XP_003598700.1\| Serine/threonine protein kinase [Medicago truncatula] | 7.00E-07 |
| gene=XLOC_015142 | >ref\|XP_003550174.1\| PREDICTED: monothiol glutaredoxin-S2-like [Glycine max] | 3.00E-49 |
| gene=XLOC_011074 | >ref\|XP_003543162.1\| PREDICTED: carbon catabolite repressor protein 4 homolog 5-like isoform X1 [Glycine max] | 2.00E-04 |
| gene=XLOC_003911 | >ref\|XP_003529624.1\| PREDICTED: mitochondrial arginine transporter BAC2-like [Glycine max] | 3.00E-04 |
| gene=XLOC_004510 | >ref\|XP_006577118.1\| PREDICTED: cohesin subunit SA-1 isoform X2 [Glycine max] | 4.00E-12 |

| | | |
|---|---|---|
| gene=XLOC_021243 | >ref\|XP_008450744.1\| PREDICTED: sec-independent protein translocase protein TATA. | 2.00E-31 |
| gene=XLOC_001433 | >ref\|XP_006574043.1\| PREDICTED: putative ribonuclease H protein At1g65750-like [Glycine max] | 9.00E-20 |
| gene=XLOC_019818 | >ref\|XP_010647371.1\| PREDICTED: protein FAR1-RELATED SEQUENCE 5-like [Vitis vinifera] | 8.00E-12 |
| gene=XLOC_019819 | >ref\|XP_010644068.1\| PREDICTED: protein FAR1-RELATED SEQUENCE 5-like [Vitis vinifera] | 6.00E-129 |
